# Simultaneous Photodynamic Eradication of Tooth Biofilm and Tooth Whitening with an Aggregation‐Induced Emission Luminogen

**DOI:** 10.1002/advs.202106071

**Published:** 2022-05-07

**Authors:** Meijia Gu, Susu Jiang, Xiaoyu Xu, Ming‐Yu Wu, Chao Chen, Yuncong Yuan, Qingrong Chen, Yidan Sun, Luojia Chen, Chao Shen, Peng Guo, Shujie Liu, Engui Zhao, Shi Chen, Sijie Chen

**Affiliations:** ^1^ Key Laboratory of Combinatorial Biosynthesis and Drug Discovery Ministry of Education Department of Gastroenterology Zhongnan Hospital of Wuhan University and School of Pharmaceutical Sciences Wuhan University Wuhan Hubei 430079 China; ^2^ School of Science Harbin Institute of Technology, Shenzhen HIT Campus of University Town Shenzhen 518055 China; ^3^ Ming Wai Lau Centre for Reparative Medicine Karolinska Institutet Hong Kong 999077 China; ^4^ Department of Burn and Plastic Surgery Biomedical Research Center Shenzhen Institute of Translational Medicine Health Science Center Shenzhen Second People's Hospital The First Affiliated Hospital of Shenzhen University Shenzhen 518035 China; ^5^ Yanling Taocheng health center Xuchang 461226 China

**Keywords:** aggregation‐induced emission, biofilm, photodynamic therapy, Streptococcus mutans, tooth whitening

## Abstract

Dental caries is among the most prevalent dental diseases globally, which arises from the formation of microbial biofilm on teeth. Besides, tooth whitening represents one of the fastest‐growing areas of cosmetic dentistry. It will thus be great if tooth biofilm eradication can be combined with tooth whitening. Herein, a highly efficient photodynamic dental therapy strategy is reported for tooth biofilm eradication and tooth discoloration by employing a photosensitizer (DTTPB) with aggregation‐induced emission characteristics. DTTPB can efficiently inactivate *S. mutans*, and inhibit biofilm formation by suppressing the expression of genes associated with extracellular polymeric substance synthesis, bacterial adhesion, and superoxide reduction. Its inhibition performance can be further enhanced through combined treatment with chlorhexidine. Besides, DTTPB exhibits an excellent tooth‐discoloration effect on both colored saliva‐coated hydroxyapatite and clinical teeth, with short treatment time (less than 1 h), better tooth‐whitening performance than 30% hydrogen peroxide, and almost no damage to the teeth. DTTPB also demonstrates excellent biocompatibility with neglectable hemolysis effect on mouse red blood cells and almost no killing effect on mammalian cells, which enables its potential applications for simultaneous tooth biofilm eradication and tooth whitening in clinical dentistry.

## Graphical abstract

In this work, an aggregation‐induced‐emission‐active photosensitizer, DTTPB, with high reactive oxygen species sensitizing efficiency and broad absorption band is employed for photodynamic killing of planktonic bacteria, inhibition of biofilm formation, and eradication of biofilm. Besides, DTTPB‐mediated photodynamic therapy could efficiently whiten both colored oral tooth models and clinical teeth without destroying tooth structure.

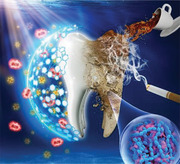

## 1 Introduction

Dental caries (tooth decay) represents one of the most common and costly chronic diseases, causing unbearable suffering in 2.3 billion people around the world.^[^ ^1^, ^2^, ^3^, ^4^ ^]^ The development of dental caries is a long and complex process, which depends on the formation of microbial biofilms on tooth surfaces (dental plaque).^[^ ^4^, ^5^, ^6^ ^]^ The formation of biofilms on tooth can increase the risk of enamel decalcification and periodontal diseases. To prevent the development of gingivitis, caries, and periodontal diseases, cutting off biofilm formation is an effective method. Scientists have devoted lots of research effort to interfering with the growth and metabolism of bacteria in the plaque biofilms, and preventing the formation or promotion of the dissociation of microbial biofilms, in hope of preventing dental caries.

*Streptococcus mutans* (*S*. *mutans*) is a predominant etiological agent of dental caries with the exceptional capability of acid production and biofilm formation.^[^ ^7^, ^8^, ^9^ ^]^ There are multiple mechanisms of *S. mutans* contributing to the formation and development of dental plaque biofilm, including extracellular polymeric substance (EPS) synthesis, carbon catabolite repression, and quorum sensing.^[^ ^10^, ^11^ ^]^ The EPS matrix of *S. mutans* is a water‐insoluble, 3D natural physical barrier, which protects *S. mutans* from its host's innate immune cells and prevents the penetration of antibacterial agents.^[^ ^12^, ^13^ ^]^ Burying bacteria cells in biofilms is an ecological strategy that bacteria adopt to get rid of the host immune system and antimicrobial drugs, and the biofilms serve as a reservoir for chronic infections with high severity.^[^ ^14^, ^15^, ^16^ ^]^ Besides, bacteria inside biofilm are usually metabolically changed with low growth rates and increased stress resistance, which invalidates antimicrobials and increases the difficulties of eradicating these bacteria.^[^ ^15^, ^16^, ^17^ ^]^

To inhibit the formation of biofilm, most commercially available oral care products contain ingredients for dental caries prevention, such as chlorhexidine (CHX) and fluoride. However, both of them cannot modulate biofilm composition or its virulence, and may lead to tooth staining.^[^ ^18^ ^]^ Even with regular use of fluoride, carious lesions could still develop when exposed to over six dietary sugar per day, while high dose of fluoride could be associated with fluorosis, bone weakening, and developmental neurotoxicity. CHX has been reported to be toxic to host cells and may induce allergic reactions. More importantly, microorganisms have gained increasing resistance to these antimicrobials, which necessitates novel tooth biofilm eradication and tooth discoloration strategies to which microorganisms are difficult to generate resistance.

Besides biofilm eradication, there is an increasing need for good tooth appearance. The color of teeth can be significantly altered by stains from various sources, such as smoking, consumption of tannin‐rich beverages (e.g., tea), and abuse of antibacterial agents such as CHX. Tooth whitening has thus emerged as one of the most demanded dental treatments by the general public. Compared with irreversible therapies such as veneers, tooth whitening represents a conservative and convenient treatment for colored teeth.^[^ ^19^ ^]^ During regular tooth whitening, hydrogen peroxide (H_2_O_2_) and carbamide peroxide are widely used for the removal of tooth stains in the form of whitening strips (a tooth discoloration product). Most whitening strips contain over 10% of H_2_O_2_, whose bleaching effect is similar to that of household tray bleaching agents.^[^ ^20^ ^]^ Such caustic treatment can cause burns to gingival and mucosal tissues,^[^ ^21^ ^]^ and may also lead to dentine hypersensitivity.^[^ ^22^ ^]^ It has been reported that H_2_O_2_ not only destroys the morphology and reduces the hardness of enamel,^[^ ^23^ ^]^ but also increases surface porosities, which may lead to tooth re‐coloration and adherence of certain cariogenic microorganisms on teeth.^[^ ^24^ ^]^

As aforementioned, both tooth biofilm inhibition and tooth whitening are thorny issues, and it will be great if we can tackle these two issues in one combined treatment. Photodynamic therapy (PDT) has been recently proposed for applications in dental therapies and tooth whitening, and it has been considered as a promising strategy to eradicate oral pathogenic bacteria, which may cause endodontic diseases, periodontitis, and caries.^[^ ^25^, ^26^, ^27^, ^28^, ^29^, ^30^ ^]^ During the course of PDT, photosensitizers (PSs) and light irradiation were employed to sensitize the generation of reactive oxygen species (ROS), which can cause oxidative damage to nucleic acids, proteins, and lipids.^[^ ^31^ ^]^ Consequently, it induces irreversible microbial death with the superiority of minimal invasiveness, limited antibiotic resistance, low systemic toxicity, and minimal side effects. Recently, Zhang et al. reported a bifunctional photodynamic dental therapy strategy for tooth whitening and biofilm eradication by employing a zwitterion‐modified porphyrin as the PS.^[^ ^32^ ^]^ However, high PS concentrations, long incubation time, and long irradiation time were needed to achieve a good tooth‐whitening effect and bactericidal effect in biofilm, which may increase the discomfort of patients and limit its clinical applications. Besides, conventional PSs usually suffer from decreased ROS sensitizing efficiency at high concentrations and in aggregated state, and thus further increasing their working concentrations may not improve PDT performance.

**Figure 1:**
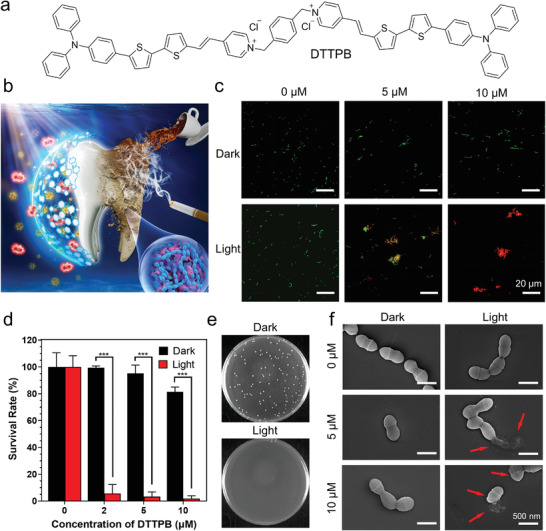
Photodynamic antibacterial effect of DTTPB. a) Molecular structure of DTTPB. b) Schematic illustration of the simultaneous photodynamic eradication of tooth biofilm and tooth whitening processes with DTTPB. c) Evaluation of the viable state of *S. mutans* by using a live & dead viability/cytotoxicity assay kit (US Everbright INC.). *S. mutans* cells were pretreated without/with 10 µm of DTTPB, followed by storage in dark or white‐light irradiation (36 mW cm^−2^) for 10 min. Afterwards, live & dead viability/cytotoxicity assay kit was employed to determine the viable state of *S. mutans*. 488 nm laser and 515–550 nm emission filter were used for the green channel, while 561 nm laser and 570–620 nm emission filter were used for the red channel. d) *S. mutans* survival rate evaluated by serial dilution test on BHI agar. *S. mutans* were treated without/with varied concentrations of DTTPB, followed by storage in dark or white‐light irradiation (36 mW cm^−2^) for 10 min. Data are presented as mean ± standard deviation with at least three replications. e) Representative images of BHI agar plates employed for quantification of *S. mutans* viability. Both groups were treated with 10 µm of DTTPB. f) Morphology study of *S. mutans* cells. *S. mutans* were incubated with designated concentrations of DTTPB, followed by storage in dark or irradiating with white light (36 mW cm^−2^) for 10 min.

Materials with aggregation‐induced emission (AIE) characteristics are gaining increasing attention, and they have been employed as PSs for microorganisms and tumor treatments due to their superior advantages of good photostability and enhanced ROS production in the aggregated state.^[^ ^33^, ^34^, ^35^, ^36^, ^37^, ^38^, ^39^ ^]^ Previously, we reported an AIE‐active PS, DTTPB (**Figure** **1**a), for efficient inactivation of human coronavirus with PDT.^[^ ^40^ ^]^ Encouraged by these exciting results, we further explored its potential for simultaneous photodynamic eradication of biofilm and tooth whitening (Figure 1b). DTTPB can sensitize the production of ROS, effectively inactivate *S. mutans* both in planktonic solution and biofilm, inhibit the formation of biofilm, and disintegrate biofilm by decomposing exopolysaccharides and glycoproteins in the EPS matrix. Besides, it can also whiten colored saliva‐coated hydroxyapatite (sHA) and clinical teeth at low working concentrations and within a short irradiation time without inducing tooth erosion. Such highly efficient simultaneous tooth biofilm eradication and tooth whitening of DTTPB‐mediated PDT is expected to open up new avenues for clinical oral treatment practices.

## 2 Results and Discussions

### 2.1 Photodynamic Killing of Planktonic *S. mutans* with DTTPB

DTTPB was synthesized following the procedures reported previously.^[^ ^40^ ^]^ DTTPB is featured with a D‐*π*‐A structure, with triphenylamine groups and bithiophene serving as an electron donor, a carbon‐carbon double bond functioning as the *π*‐bridge, and a pyridinium group acting as an electron acceptor. DTTPB bears a positively charged head and two hydrophobic tails, which mimics the structure of phospholipids on biological membranes, and is expected to interact with bacterial cell envelopes through both electrostatic interactions and hydrophobic interactions. We first incubated both Gram‐positive (*S. mutans* and *S. aureus*) and Gram‐negative (*E. coli*) bacteria with DTTPB, and all the three tested bacteria could be stained by DTTPB (Figure S1, Supporting Information), which proved the strong interactions of DTTPB towards bacteria. As *S. mutans* was the dominant pathogenic bacteria of tooth biofilm, it was employed for further studies. The interactions of DTTPB with bacteria could also be revealed by measuring the photoluminescence (PL) spectra of DTTPB in the absence/presence of *S. mutans*. DTTPB were incubated with *S. mutans* in PBS, and the fluorescence spectra of these solutions were collected. Besides, the fluorescence spectra of DTTPB in PBS and PBS alone were also recorded for comparison. As shown in Figure S2, Supporting Information, DTTPB emitted faintly in PBS. The fluorescence intensity of DTTPB could be increased by the presence of *S. mutans*, suggesting the binding of DTTPB towards *S. mutans*. Upon binding to *S. mutans*, the fluorescence of DTTPB was peaked at 650 nm, which was almost the same as that in the presence of DOPC,^[^ ^40^ ^]^ the major component of membrane structures. This proved the binding of DTTPB towards the membrane structure of *S. mutans*.

In PDT, good ROS sensitizing ability of the PSs is crucial for achieving a good therapeutic effect. DTTPB with a D‐*π*‐A structure was expected to possess low energy gap between its singlet excited states and triplet excited states, which could promote intersystem crossing and thus enhance ROS sensitizing efficiency. As ^1^O_2_ was considered the first ROS generated during PDT, we evaluated the ^1^O_2_ sensitizing ability of DTTPB by employing 9,10‐anthracenediyl‐bis(methylene)dimalonic acid (ABDA) as the indicator.^[^ ^41^ ^] 1^O_2_ could react with ABDA and result in a decrease in absorbance of ABDA. As illustrated in Figure S3, Supporting Information, the absorbance of ABDA (50 mm) at 378 nm in the solution containing DTTPB (20 µm) was decreased to 1% of its original level upon white‐light irradiation (36 mW cm^−2^) for 5 min, while the absorption of ABDA alone was almost not changed under the same experimental conditions. This substantially demonstrated the excellent ^1^O_2_ sensitizing ability of DTTPB. Besides, Rose Bengal was employed for comparison. DTTPB demonstrated higher ROS sensitizing efficiency than Rose Bengal, suggesting that DTTPB was an excellent sensitizer for PDT.

As one of the dominant pathogenic bacteria of tooth biofilm, *S. mutans* usually leads to irreversible dental caries,^[^ ^42^ ^]^ and inactivation of planktonic *S. mutans* is the primary requirement for removal of tooth biofilm. We thus evaluated the PDT effect of DTTPB on planktonic *S. mutans* with a live & dead viability/cytotoxicity assay kit (US Everbright INC.). In this bacterial viability kit, NucGreen stained alive bacteria, and endowed them with green fluorescence, while EthD‐III was cell membrane impermeable and only stained dead bacteria with compromised membranes. As shown in Figure 1c, when *S. mutans* was incubated with DTTPB in dark, only green fluorescence from DTTPB was observed, suggesting these bacteria were still alive. With light irradiation alone, the bacteria were still alive with green fluorescence. If the bacteria were stained with DTTPB, and then treated with light irradiation (36 mW cm^−2^), red emission of EthD‐III could be discerned from the bacteria, which proved the killing effect of DTTPB towards *S. mutans*. At a DTTPB concentration of 10 µm, almost all the bacteria exhibited bright red fluorescence, demonstrating the good PDT performance of DTTPB towards *S. mutans*. The survival rates of *S. mutans* were determined by the plate‐count method (Figure 1d,e and Figure S4, Supporting Information). Without light irradiation, no antibacterial effect was observed, while nearly 95%, 97%, and 99% of *S. mutans* were killed after treatment with light irradiation and 2, 5, and 10 µm of DTTPB, respectively (Figure 1d). The antibacterial effect was confirmed by spot plate assay (Figure S5, Supporting Information). Besides, scanning electron microscope (SEM) was also employed to investigate the PDT process of DTTPB (Figure 1f). Without light irradiation, *S. mutans* showed intact and smooth surfaces both in the absence and presence of DTTPB, while a void was observed on irradiated *S. mutans*. This suggested that DTTPB‐mediated PDT led to the disruption of bacterial membranes, and induced the extrusion of the intracellular content. These observations unambiguously revealed the direct destroying effect of ROS sensitized by DTTPB. Thanks to the membrane targeting and high ROS sensitizing efficiency of DTTPB, it demonstrated satisfying bactericidal performance.

### 2.2 DTTPB‐Mediated PDT Inhibited Biofilm Formation and Acid Production

**Figure 2:**
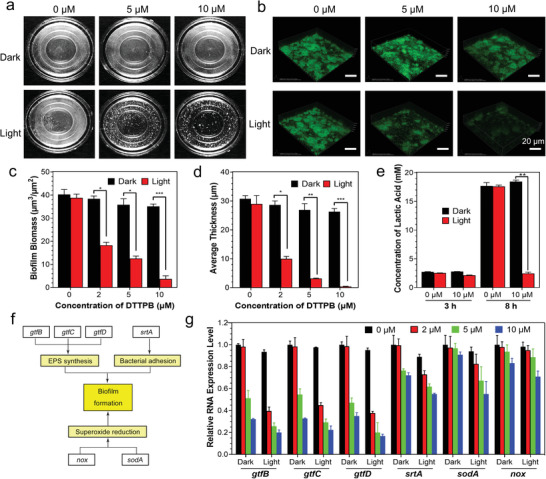
Photodynamic inhibition of biofilm formation by DTTPB. a) Images of biofilms formed on culture dish by *S. mutans* after treatment without/with DTTPB and light irradiation (36 mW cm^−2^) for 10 min. b) Confocal images of the biofilms formed by *S. mutans*. *S. mutans* were treated without/with DTTPB and light irradiation (36 mW cm^−2^) for 10 min, cultured for one day, and then stained with NucGreen for 15 min. 488 nm laser and 515–550 nm emission filter were used for *S. mutans* biofilm imaging. c–e) Photodynamic effect of DTTPB on (c) biofilm biomass, (d) average thickness, and (e) lactic acid production of *S. mutans* biofilm. Quantification of (c) biofilm biomass, and (d) average thickness of live bacteria were calculated according to 5 random sights of *S. mutans* biofilms by COMSTAT. For evaluation of lactic acid production, *S. mutans* were treated without/with 10 µm of DTTPB and light irradiation (36 mW cm^−2^) for 10 min. Data are displayed as mean ± standard deviation. f) Schematic illustration of the biological functions of *gtfB*, *gtfC*, *gtfD*, *srtA*, *nox*, and *sodA* genes. g) Relative RNA expression of *gtfB*, *gtfC*, *gtfD*, *srtA*, *nox*, and *sodA* genes without/with DTTPB and white‐light irradiation (36 mW cm^−2^) treatment. Data are shown as mean ± standard deviation with three replications.

As *S. mutans* is the predominant etiological agent for biofilm formation on tooth, DTTPB with a good killing effect on *S. mutans* is expected to greatly inhibit biofilm formation process. That is really the case: when *S. mutans* was pretreated with DTTPB‐mediated PDT, its biofilm formation was greatly inhibited, while the *S. mutans* treated with DTTPB alone or light irradiation alone could still form biofilms under the same experimental conditions (**Figure** **2**a). To further assess the DTTPB‐mediated PDT processes, the biofilms formed with different pretreatments were stained with NucGreen and imaged under confocal fluorescence microscopy combined with quantitative computational analysis. Each slice of the biofilm was scanned and the 3D images are shown in Figure 2b. When *S. mutans* were pretreated with only light irradiation or DTTPB, bright green fluorescence of NucGreen was detected, suggesting the large quantity of *S. mutans* reproduced and the successful formation of biofilms. When *S. mutans* were treated with both light irradiation and DTTPB, the fluorescence from NucGreen was substantially decreased, suggesting the low numbers of *S. mutans*. According to the 3D fluorescence images of the biofilms, biofilm biomass and average thickness of live bacteria were calculated (Figure 2c,d) by employing Comstat2, which clearly revealed the decreased biofilm biomass and thickness after DTTPB‐mediated PDT. These results suggested that after DTTPB‐mediated PDT, the biofilm formation ability of *S. mutans* was substantially decreased. The results were in good accordance with the bactericidal performance of DTTPB‐mediated PDT.

Once biofilms are formed, pathogens, such as *S. mutans* and other cariogenic bacteria, could embed in EPS and produce highly acidic microenvironments with pH values close to 4.5, which may lead to the onset of dental caries.^[^ ^43^ ^]^ Thus, we assessed the effect of DTTPB‐mediated PDT on acid production of *S. mutans* by measuring the lactic acid content in *S. mutans* cultures (Figure 2e). The lactic acid production of *S. mutans* cells was significantly inhibited by DTTPB, which suggested that DTTPB‐mediated PDT was very effective in preventing dental caries.

To gain deeper insight into the underlying mechanisms of DTTPB‐mediated PDT on inhibiting biofilm formation, we determined the expression levels of biofilm‐related genes in *S. mutans* (Figure 2f). *gtfB*, *gtfC*, and *gtfD* genes are related to EPS synthesis. By encoding three critical glucosyltransferase (GTF) enzymes, GtfB, GtfC, and GtfD, which are associated with glucan productions in *S. mutans*, *gtfB*, *gtfC*, and *gtfD* genes could modulate the formation and development of *S. mutans* biofilm.^[^ ^44^ ^]^ The expression levels of these genes are thus closely associated with the production of EPS. To quantify the expression of these genes, *S. mutans* were treated with different concentrations (0, 2, 5, and 10 µm) of DTTPB followed by storage in the dark or light irradiation. Afterwards, their total RNA was extracted and the target genes were quantified with qRT‐PCR by using 16S rRNA as an internal reference (Table S1, Supporting Information). As illustrated in Figure 2g, treatment with DTTPB downregulated the expression of these three genes in a dose‐dependent manner. When the concentration of DTTPB was 2 µm, its inhibitory effect on light‐irradiated group was significantly stronger than that of the dark group. This suggested that ROS production could suppress the expression of these genes. As the concentration of DTTPB was increased, both light‐irradiated groups and dark‐treated groups exhibited low expression levels of these three genes, which was indicative of the direct suppression effect of DTTPB in the dark. DTTPB may interact with the components in cell envelopes and regulate the expression of these genes. For comparison, the expression levels of these three genes in *S. mutans* treated with white‐light irradiation alone were also examined, and the results indicated that light irradiation alone could not suppress the expression of these genes, which was in good accordance with the antibacterial experiments.

*srtA* gene is associated with bacterial adhesion. Its trend corresponding to DTTPB‐mediated PDT was similar to that of *gtf* genes, suggesting that DTTPB‐mediated PDT could inhibit *S. mutans* from bonding with each other and formation of biofilms. *sodA* gene encodes superoxide dismutase (SODs), which is an important virulence factor that reduces superoxide,^[^ ^45^ ^]^ and thus decreases hydroxyl level.^[^ ^46^ ^]^ Incubating *S. mutans* in the dark did not significantly downregulate *sodA* expression, however, there was a dose‐dependent dowregulation of *sodA* expression in *S. mutans* treated with both DTTPB and light irradiation. *Nox* could direct the production of an oxygen‐dependent NADH oxidase, and is involved in the conversion of NADH to NAD^+^, which is essential for a non‐stop glycolysis to generate pyruvate. The *nox* gene expression level in DTTPB‐treated (10 µm) *S. mutans* was obviously reduced as compared with the control group. The above results indicated DTTPB‐mediated PDT inhibited biofilm formation through downregulating the expression of oxidative stress‐related genes, which increased the sensitivity of *S. mutans* towards ROS and thus elevated the PDT performance.

Collectively, besides directly damaging biomolecules of *S. mutans* through PDT, DTTPB and the ROS it sensitized could also downregulate genes associated with EPS synthesis, bacterial adhesion, and superoxide reduction, which could joint force together and inhibit the formation of biofilms by *S. mutans*.

### 2.3 DTTPB‐Mediated Photodynamic Eradication of Biofilm and PDT Combined Treatment with Chlorhexidine in In Vitro Oral Model

**Figure 3:**
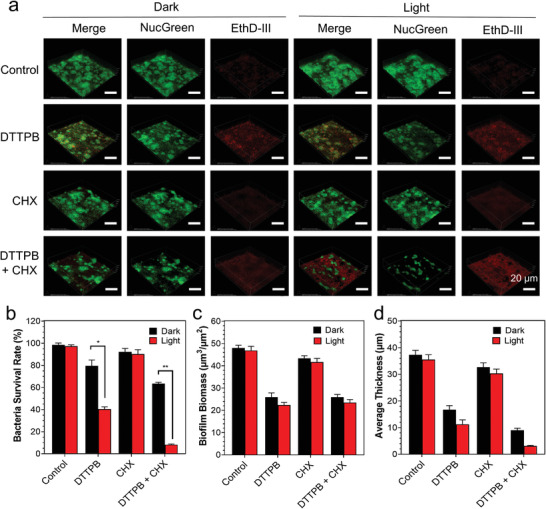
Combination of PDT and CHX for highly effective eradication of *S. mutans* biofilm in in vitro oral models on glass dishes. a) Delegate 3D images of biofilms treated without/with DTTPB and white‐light irradiation. 1‐day‐old *S. mutans* biofilms were co‐cultured without/with 20 µm of DTTPB and white‐light irradiation for 10 min (36 mW cm^−2^), treated without/with 0.12% CHX for 30 min, followed by staining with live & dead viability/cytotoxicity assay kit (US Everbright INC.) 488 nm laser and 515–550 nm emission filter were employed the green channel. 561 nm laser and 570–620 nm emission filter were used for the red channel. b) Quantification of survival rate according to the ratio of red/green fluorescence intensity. c,d) Quantification of (c) biofilm biomass, and (d) average thickness of live bacteria according to 5 random sights of *S. mutans* biofilms by COMSTAT2. Data are displayed as mean ± standard deviation.

Besides inhibiting the formation of biofilm, DTTPB‐mediate PDT could also eradicate existing biofilms. DTTPB could stain biofilm and endow biofilm with red fluorescence (Figure S6, Supporting Information), which further enabled its eradication of existing biofilm. To demonstrate this, we first verified the ROS sensitizing ability of DTTPB in biofilms and a commercially available probe for ROS, dichlorodihydrofluorescein diacetate (DCFH), was employed to detect the ROS. Due to the absence of large conjugation, DCFH is not fluorescent. Reacting with ROS could produce a fluorescein core in DCFH and turn it into an emissive form. As shown in Figure S7a, Supporting Information, without DTTPB, no discernable fluorescence was detected from biofilms incubated with DCFH. When the biofilm was treated with 20 µm of DTTPB in dark, very dim fluorescence of DCFH could be detected. In the presence of both DTTPB and light irradiation, bright green emission from DCFH was clearly observed. The fluorescence of DCFH in light‐ and DTTPB‐treated biofilm was around 7.5 times higher than that of the biofilm treated with only DTTPB (Figure S7b, Supporting Information), which proved the ROS sensitizing ability of DTTPB in biofilm. Afterwards, the performance of DTTPB‐mediated PDT towards *S. mutans* embedded in biofilms was investigated following the reported methods.^[^ ^47^ ^]^ To mimic a pathogenic situation, biofilms were formed on glass in the presence of sucrose, which acted as the substrate for EPS synthesis and acid production. The as‐prepared biofilm was irradiated with white light for 10 min in the presence of 20 µm DTTPB. Afterwards, the biofilm was rinsed three times with PBS, incubated with live & dead viability/cytotoxicity assay kit (US Everbright INC.), and then subjected to fluorescence imaging with a laser scanning confocal microscope. The 3D images of the films are shown in **Figure** **3**a. Without DTTPB or light irradiation, only green fluorescence of NucGreen was observed, suggesting the viable state of these bacteria. Upon treatment with both DTTPB and light irradiation, red fluorescence of EthD‐III was detected, which was indicative of the presence of dead bacteria. For comparison, the destroying effect of the main effective component of mouthwashes, CHX, was also investigated. CHX is a cationic compound that interacts with the negative charges of bacterial cell walls, leading to the destabilization of bacterial membranes. After treating the as prepared biofilm with 0.12% CHX for 30 min, neglectable killing effect was observed both in the absence and presence of white‐light irradiation, with strong green fluorescence of NucGreen and only dim red fluorescence of EthD‐III in the bacterial viability test. This result was consistent with the previous report that oral CHX treatment at the clinical concentration of 0.12% for 24 h could not destroy biofilm effectively.^[^ ^48^ ^]^ We also combined DTTPB‐mediated PDT and CHX treatment to check their synergetic effect. The combined treatment demonstrated even better performance than DTTPB‐mediated PDT and CHX treatment alone, with green fluorescence of NucGreen in only a few spots and strong red fluorescence of EthD‐III.

To gain a quantitative view of these treatments, bacterial survival rate, biofilm biomass, and average thickness of the biofilm were deduced through statistical analysis of the fluorescence intensity and distribution (Figure 3b–d). Upon treatment with DTTPB‐mediated PDT, the bacterial survival rate dropped to around 40% (Figure 3b), which suggested that DTTPB‐mediated PDT killed bacteria efficiently in tooth biofilm. Besides, after DTTPB‐mediated PDT treatment, the biofilm biomass (Figure 3c) and biofilm thickness (Figure 3d) were also decreased, suggesting that it also destroyed the biofilm and reduced biofilm thickness. In contrast, CHX treatment only led to slight decreases in the bacterial survival rate, biofilm biomass, and average thickness, which was indicative of its poor performance in the eradication of biofilms. The combination of DTTPB‐mediated PDT and CHX resulted in a low bacterial survival rate of 8.3% and a significant loss of average thickness, manifesting the excellent biofilm eradication performance of this combined treatment. The biofilm eradication effect was also confirmed by Crystal Violet staining (Figure S8, Supporting Information), which showed a similar trend as the biomass. These results proved that DTTPB was very efficient for PDT eradication of biofilms, and its combined usage with CHX could further promote its performance. Such good PS as DTTPB with good ROS sensitizing efficiency and biofilm eradication performance was ideal candidate for photodynamic dental applications.

**Figure 4:**
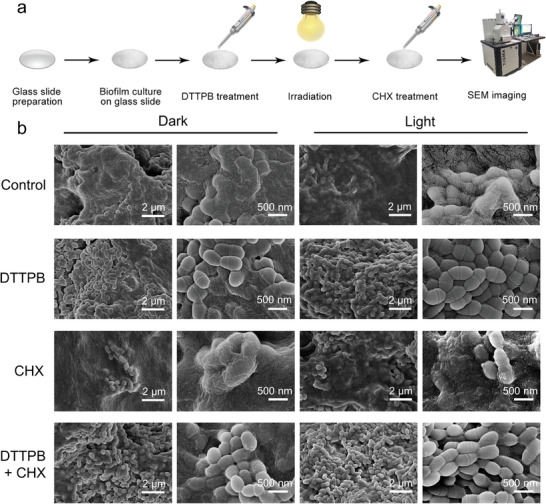
a) Schematic illustration of experimental processes for determining the morphology changes of *S. mutans* biofilms after combined treatment with DTTPB and CHX on glass. b) SEM images of *S. mutans* biofilms. 1‐day‐old *S. mutans* biofilms on glass were cultured without/with 20 µm of DTTPB and white‐light irradiation for 10 min (36 mW cm^−2^), and treated without/with 0.12% CHX for 30 min.

To uncover the origin of DTTPB‐mediated PDT for biofilm eradication, the morphology of bacteria with different treatments was investigated by SEM. The experimental procedures for SEM imaging are shown in **Figure** **4**a. To obtain biofilm with good adherence, glass substrates were pretreated with saliva following reported procedures.^[^ ^48^ ^]^ Afterwards, these glass substrates were immersed in BHI media containing 1wt% sucrose and *S. mutans*. After culturing for one day, biofilms were formed on the glass substrates. Once the biofilm was formed, it was subjected to DTTPB‐mediated PDT treatment and CHX treatment, followed by SEM imaging (Figure 4b). Without DTTPB‐mediate PDT and CHX treatment, *S. mutans* were bounded together in biofilms by EPS, and *S. mutans* envelops were intact with regular rod‐shapes. Upon incubation with DTTPB, the EPS of *S. mutans* biofilms were greatly decreased, and *S. mutans* could be differentiated from each other with clear edges. Interestingly, even the biofilm without light‐irradiation treatment showed less EPS than the control group. In the pictures with higher magnifications, *S. mutans* with deformed morphology were observed in DTTPB‐treated biofilms. The SEM images of CHX‐treated biofilms were similar to that of the control group, suggesting its vainness in promoting the degradation of EPS. For the biofilm with combined treatment of DTTPB‐mediated PDT and CHX, the outlines of the *S. mutans* cells were clearly discernable with neglectable EPS. The results demonstrated that the combined treatment of DTTPB‐mediated PDT and CHX could not only kill the *S. mutans*, but also degrade the EPS. In this process, DTTPB sensitized the production of ROS, which could oxidize both cellular components and EPS, and induce antibacterial effect and biofilm degradation effect, respectively. Besides, the amphiphilic nature of DTTPB enabled it to serve as a detergent to penetrate into the hydrophobic regions of EPS, which was beneficial for biofilm degradation. SEM characterizations were also performed on in vitro oral model, in which saliva‐coated hydroxyapatite (pellicle‐coated tooth mimetics) sHA disk was employed as the substrate to simulate tooth. Similar morphology trends were observed (Figure S9, Supporting Information).

### 2.4 Highly Efficient and Non‐Destructive Tooth Discoloration with DTTPB

**Figure 5:**
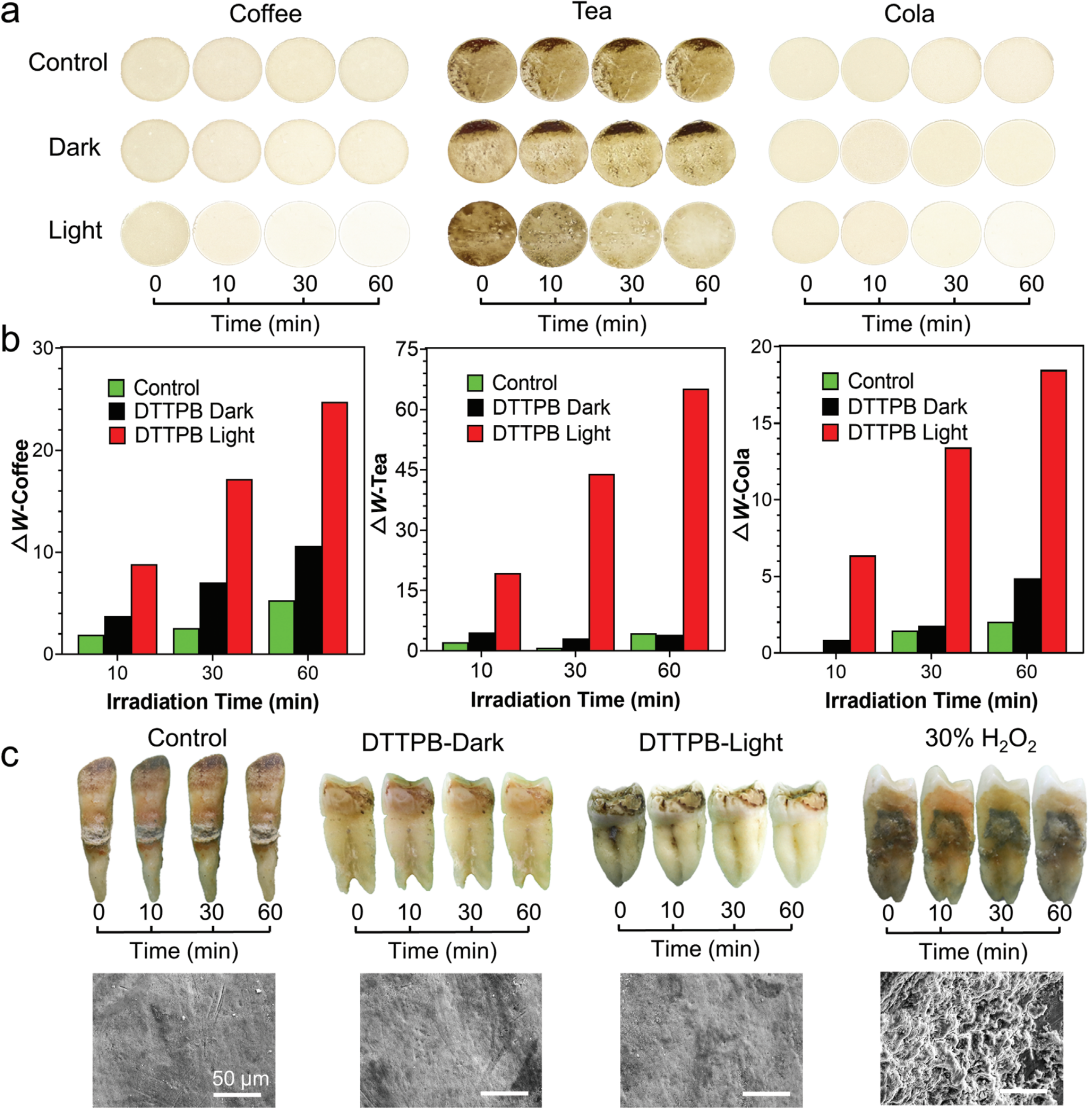
Whitening effect of DTTPB. a) Images of coffee‐, tea‐ and cola‐colored sHA treated without/with 20 µm of DTTPB and white‐light (36 mW cm^−2^) irradiation for 10, 30, and 60 min, respectively. b) Δ*W* of colored sHA treated with 20 µm of DTTPB and white‐light irradiation (36 mW cm^−2^). c) Images of clinical teeth treated with 20 µm of DTTPB without/with white light (36 mW cm^−2^) irradiation or 30% H_2_O_2_ for 10, 30, and 60 min, respectively and the corresponding SEM images of tooth surfaces after 60 min treatment.

In clinical tooth‐whitening, H_2_O_2_ is employed for oxidizing the stains. Since H_2_O_2_ is one of the ROS in PDT, it could be envisioned that DTTPB with high photosensitizing ability may be applicable to tooth whitening. Motivated by this idea, we explored the potential of DTTPB for tooth whitening. We first tested the feasibility of the whitening effect on simulated teeth (sHA). sHA were immersed in different beverages (cola, coffee, and black tea) for 7 days to simulate colored teeth. Afterwards, this sHA was subjected to DTTPB and white‐light irradiation/storage in dark. Since DTTPB was dissolved in DMSO, the control group was treated with 0.1% of DMSO in dark. In the control group, the color of sHA remained unchanged after white‐light irradiation for 60 min, while with DTTPB‐mediated PDT, the sHA became significantly whiter than its initial state (**Figure** **5**a). Interestingly, sHA treated with DTTPB alone were discernably whitened, suggesting that DTTPB in dark could promote tooth whitening. To quantitatively measure the degree of sHA whitening, 3D CIE Lab color system created by “Commission Internationale de L'Eclairae, CIE”^[^ ^49^ ^]^ was employed to process these images, which produced three parameters (*a*, *b*, and *L*) for each image (Figures S10–S12, Supporting Information). *a* and *b* were chromaticity axes, which were mutually perpendicular to each other, and were related to saturation and hue of red‐green and yellow‐blue parameters, respectively. *L* was a third axis, which denoted the lightness, and was located perpendicularly to the color plane. Following the previous report, a new parameter, whiteness (*W*), was defined as *W* = *L* − |*a*| − |*b*|).^[^ ^32^ ^]^ The changes of *W* were employed to evaluate the tooth whitening effect. As shown in Figure 5b, DTTPB‐mediated PDT induced pronounced changes of *W* of coffee‐ tea‐ and cola‐colored sHAs, which was indicative of its excellent tooth whitening performance.

We also tested the tooth whitening effect of DTTPB‐mediated PDT on the clinical tooth. Intact and noncarious teeth were collected and subjected to DTTPB treatment without/with white‐light irradiation, and photographs were taken at irradiation times of 0, 10, 30, and 60 min, respectively (Figure 5c). For comparison, 0.1% DMSO was added as the negative control and 30% H_2_O_2_ was employed as the positive control. In control groups, the color of the tooth remained unchanged throughout the experiment with 0.1% DMSO treatment, and 30% H_2_O_2_ exerted limited influence on tooth color. In contrast, after DTTPB‐mediated PDT, the colored tooth became remarkably whiter (Figures S13 and S14, Supporting Information). DTTPB in dark could also whiten the tooth due to its amphiphilicity nature. For an ideal tooth whitening method, it should possess excellent colorant removal ability, but exert minimum damage to the tooth structure. In the next place, we evaluated the impact of DTTPB‐mediate tooth whitening on tooth structure by SEM. DTTPB had almost no influence on tooth structure both in dark and with white‐light irradiation (Figure 5c). In contrast, the structure of tooth treated with 30% H_2_O_2_ was obviously damaged with a rough surface, which may lead to dentine hypersensitivity and enamel damage. This was reasonable, since largely excess H_2_O_2_ molecules could react with tooth and result in a damaged tooth surface. The results suggested that DTTPB‐mediated PDT possessed obvious advantages over the commonly used H_2_O_2_‐based method. In tooth, organic protein matrix and inorganic mineral co‐exist. Tooth stains are usually extrinsic organic molecules. In the presence of high concentration of H_2_O_2_, an aggressive bleaching agent, tooth stains and the organic matrix of the tooth will be oxidized non‐selectively and harshly, thus leading to damage to the tooth structure. DTTPB whitened teeth by both acting as a detergent and sensitizing the production of ROS, which worked synergistically to remove tooth stains. Moreover, DTTPB could target biofilms and stains, and sensitize the production of ROS upon light irradiation, which achieved a localized effect on tooth stains with minimum influence on tooth structure. Therefore, DTTPB showed outstanding tooth whitening performance but was less harmful to the teeth structure. Collectively, DTTPB can whiten teeth efficiently without destructiveness, making it a potential whitening agent for clinical usage.

### 2.5 Biocompatibility

**Figure 6:**
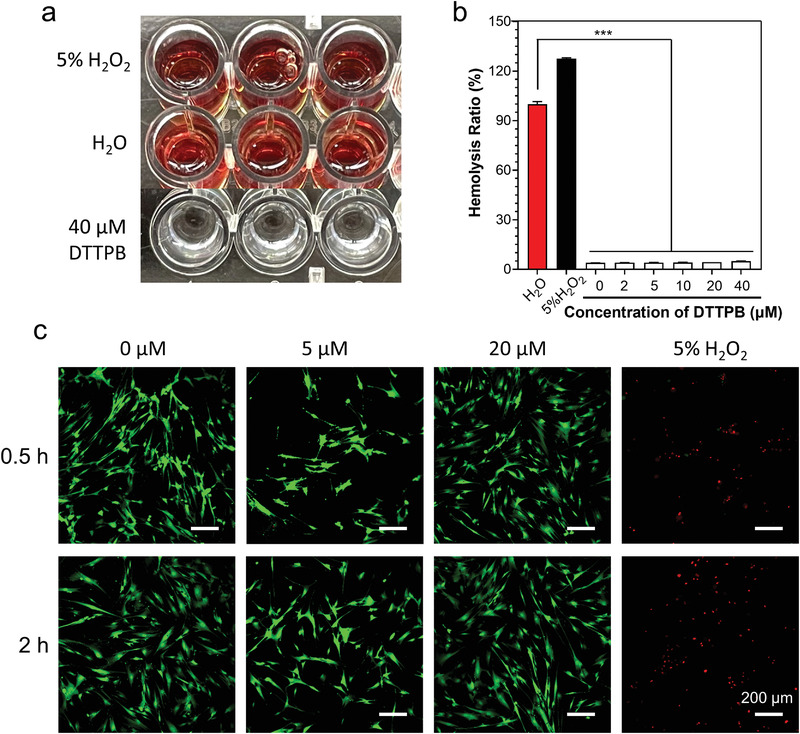
Biocompatibility of DTTPB. a,b) Evaluation of hemolysis induction by DTTPB and 5% H_2_O_2_. (b) Data are shown as mean ± standard deviation with three replications. c) Evaluation of the toxicity of DTTPB on cells. MRC‐5 cells were incubated with different concentrations of DTTPB or 5% H_2_O_2_, stained with Calcein AM/PI, followed by imaging with a confocal microscope. The images were merged from green and red channels. 488 nm laser and 500–550 nm emission filter were employed for the green channel. 561 nm laser and 570–620 nm emission filter were used for the red channel.

Previously, we demonstrated that DTTPB exerted neglectable influence on cells by employing CCK 8 experiments.^[^ ^40^ ^]^ In this work, we further evaluated its biocompatibility by conducting a hemolysis experiment and fluorescence staining. For comparison, the biocompatibility of 5% H_2_O_2_ was also evaluated under the same experimental conditions (**Figure** **6**, Figures S15 and S16, Supporting Information). As shown in Figure 6a, upon incubating fresh blood cells of mouse in deionized water, the blood cells ruptured and released its hemoglobin due to the low osmotic pressure of pure water. Thus, after centrifuging to remove red blood cells, red mixture with hemoglobin was obtained. In PBS containing 5% H_2_O_2_, the solution also became red, indicating that 5% of H_2_O_2_ could already induce the hemolysis of red blood cells. In contrast, at a DTTPB concentration of 40 µm, its PBS solution remained colorless, which was indicative of the low hemolysis ratio. By measuring the absorbance of these solutions at 540 nm, the hemolysis ratio could be calculated. As shown in Figure 6b and Figure S15, Supporting Information, based on 100% hemolysis ratio of deionized water, 5% of H_2_O_2_ led to a high hemolysis ratio of over 120%, while the hemolysis ratio of 40 µm of DTTPB was neglectable, suggesting that DTTPB was highly compatible with blood cells. We also conducted fluorescence staining to gain an intuitive idea of its cytocompatibility by employing Calcein AM and propidium iodide (PI) as indicators. Calcein AM and PI stain alive cells and dead cells, and endow them with green and red fluorescence, respectively. Thus, it will be very convenient to differentiate the dead cells from the alive cells. As shown in Figure 6c and Figure S16, Supporting Information, incubating MRC‐5 cells with up to 20 µm of DTTPB did not interfere cell viability with all MRC‐5 cell emitting green fluorescence. However, 5% of H_2_O_2_ killed almost all the cells, which were stained with PI and exhibited red fluorescence. These results clearly demonstrated the good biocompatibility of DTTPB.

## 3 Conclusions

To sum up, in this work we report the simultaneous biofilm eradication and tooth whitening with DTTPB‐mediated PDT. DTTPB was AIE active with high ROS sensitizing efficiency. It could bind to bacteria through electrostatic and hydrophobic interactions. Upon light irradiation, it sensitized the production of ROS to eliminate planktonic bacteria, such as *S. mutans* efficiently. Besides, DTTPB‐mediated PDT could also inhibit the formation of biofilm by both ROS production and downregulating genes associated with EPS synthesis (*gtfB*, *gtfC*, and *gtfD*), bacteria adhesion (*srtA*), and superoxide production (*nox* and *sodA*). Thanks to the ROS sensitizing ability and amphiphilic nature of DTTPB, it could eradicate biofilm as well, resulting in significantly decreased biofilm biomass and biofilm average thickness. The impact of DTTPB‐mediated PDT on biofilm was also investigated by SEM, which clearly revealed the EPS removal and bacteria envelop deformation effect. We also explored the potential of employing DTTPB for tooth whitening and DTTPB exhibited excellent performance at low concentration (20 µm) and in short time (60 min), with neglectable damage to teeth and excellent biocompatibility to both blood cells and human fibroblast cells. DTTPB is expected to find practical applications for simultaneous biofilm formation and tooth whitening in clinics and innovate related fields.

## Conflict of Interest

The authors declare no conflict of interest.

## Supporting information

Supporting Information

## Data Availability

Research data are not shared.
