# Supplementary material for: Simultaneous Photodynamic Eradication of Tooth Biofilm and Tooth Whitening with an Aggregation‐Induced Emission Luminogen
Source: Adv Sci (Weinh). 2022 May 7;9(20):2106071. doi: 10.1002/advs.202106071 (PMC9284169; doi:10.1002/advs.202106071)
Supplement: Supplementary file 1 — Supporting Information [file ADVS-9-2106071-s001.pdf]

ADVANCED SCIENCE
Open Access

# Supporting Information

for *Adv. Sci.*, DOI 10.1002/advs.202106071

Simultaneous Photodynamic Eradication of Tooth Biofilm and Tooth Whitening with an Aggregation-Induced Emission Luminogen

*Meijia Gu, Susu Jiang, Xiaoyu Xu, Ming-Yu Wu, Chao Chen, Yuncong Yuan, Qingrong Chen, Yidan Sun, Luojia Chen, Chao Shen, Peng Guo, Shujie Liu, Engui Zhao*, Shi Chen* and Sijie Chen**

# Supporting Information

## Simultaneous Photodynamic Eradication of Tooth Biofilm and Tooth Whitening with An Aggregation-Induced Emission Luminogen

*Meijia Gu*[1], *Susu Jiang*[1], *Xiaoyu Xu*[1], *Ming-Yu Wu*[3], *Chao Chen*[4], *Yuncong Yuan*[1], *Qingrong Chen*[1], *Yidan Sun*[1], *Luojia Chen*[1], *Chao Shen*[1], *Peng Guo*[1], *Shujie Liu*[5], *Engui Zhao*[2,*], *Shi Chen*[1,4,*] *and Sijie Chen*[3*]

[1] Key Laboratory of Combinatorial Biosynthesis and Drug Discovery, Ministry of Education, Department of Gastroenterology, Zhongnan Hostipal of Wuhan University, and School of Pharmaceutical Sciences, Wuhan University, Wuhan, 430079, Hubei, China
[2] School of Science, Harbin Institute of Technology, Shenzhen, HIT Campus of University Town, Shenzhen 518055, China
Dr. M.-Y. Wu and Dr. Sijie Chen
[3] Ming Wai Lau Centre for Reparative Medicine, Karolinska Institutet, Hong Kong, 999077, China
[4] Department of Burn and Plastic Surgery, Biomedical Research Center, Shenzhen Institute of Translational Medicine, Health Science Center, Shenzhen Second People's Hospital, the First Affiliated Hospital of Shenzhen University, Shenzhen 518035, China
[5] Yanling Taocheng health center, Xuchang, 461226, China

E-mail: zhaoengui@hit.edu.cn, shichen_2021@163.com, sijie.chen@ki.se

**1. Experimental Procedures ........................................................ S3**

1.1 Materials and general instruments ........................................................ S3

1.2 Cell and bacteria strains, and growth conditions........................................ S3

1.3 Photoluminescence measurements........................................................ S4

1.4 Bacteria culturing, staining and imaging ................................................ S4

1.5 Killing test of planktonic bacteria.......................................................... S5

1.6 Biofilm imaging ................................................................................. S6

1.7 Inhibition of biofilm formation ............................................................ S7

1.8 Oral biofilm model. ............................................................................ S7

1.9 Measurement of ROS .......................................................................... S8

1.10 Lactic acid measurement.................................................................... S9

1.11 RNA isolation and quantitative real-time PCR (qRT-PCR)........................ S10

1.12 Biofilm eradication test ...................................................................... S10

1.13 Discoloration test of DTTPB on sHA ................................................... S11

1.14 Teeth discoloration test of DTTPB........................................................ S11

1.15 Hemolysis test .................................................................................. S12

1.16 Cytotoxicity assay ............................................................................. S12

1.17 Statistical methods ............................................................................ S12

**2. Supplementary Figures ......................................................... S13**

# 1. Experimental Procedures

## 1.1 Materials and general instruments

All chemical reagents were obtained from J&K Scientific, and used without further purification. Minimum essential medium (MEM) was purchased from Gibco. Phosphate buffered saline (PBS), penicillin and streptomycin were purchased from Invitrogen. Fetal bovine serum (FBS), trypsin-EDTA, yeast extract and tryptone were obtained from Thermo Fisher Scientific. NaCl was supplied by Sinopharm Chemical Reagent. BHI was purchased from Qingdao Hope Bio-Technology Co., Ltd. Live & Dead™ Viability/Cytotoxicity Assay kit, lactic acid assay kit (E-BC-K044-M) were purchased from Elabscience, Wuhan, China. Calcein AM/PI Live & Dead™ mammalian cell viability kit was purchased from US EVERBRIGHT® INC, Suzhou, China. Chlorhexidine (CHX) was supplied by Macklin, Shanghai, China. Milli-Q water was supplied by Milli-Q Plus System (Millipore Corporation, United States). Multiska GO (Thermo Scientific) was employed to measure the photoluminescence (PL) spectra in bulk solutions, and Molecular Devices SpectraMax i3x Multi-ModeMicroplate Detection System was used to measure the PL spectra in 96-well plates. Scanning electron microscopy images were collected using a Zeiss Sigma 300. Fluorescence images were collected on a confocal laser scanning microscope (CLSM, Nikon, Eclipse Ti-S, Japan), and analyzed by using NIS-Elements AR software.

## 1.2 Cell and bacteria strains, and growth conditions

*Escherichia coli* (*E. coli*) DH10B and *Staphylococcus aureus* (*S. aureus*) ATCC 6538 were grown in LB liquid culture medium at 37 °C overnight. The concentration of bacteria was determined by measuring the optical density at 600 nm ($OD_{600}$). *Streptococcus mutans* (*S. mutans*) ATCC 25175 were kept by our lab. *S. mutans* were cultured anaerobically in BHI broth at 37 °C [1]. To enhance *S. mutans* biofilm formation, 1% (wt/vol) sucrose was added into cultures [2]. The concentration of *S. mutans* was determined by measuring the $OD_{600}$. The *S. mutans* concentration was

then adjusted to an $OD_{600}$ value of 0.6 by diluting the bacterial suspension with BHI medium. After centrifuging at 10,000 ×g for 1 min, the *S. mutans* were collected. The culture medium was then removed and the bacteria were washed three times with PBS, and then resuspended in PBS.

Human embryonic lung fibroblast cells (MRC-5) were provided by the China Center for Type Culture Collection. MRC-5 cells were cultured in MEM medium supplemented with 10% of heat-inactivated fetal bovine serum, 100 units $mL^{-1}$ penicillin and 100 μg $mL^{-1}$ streptomycin in a 5% $CO_2$ humid incubator at 37 ℃.

### 1.3 Photoluminescence measurements

*S. mutans* were cultured until the $OD_{600}$ value reached 0.6, followed by centrifuging at 10,000 ×g for 1 min. The supernatant was removed and the pellet was washed 3 times with PBS. 1 μL of DTTPB (5 mM in DMSO) was added into 1 mL PBS without/with *S. mutans* to reach a final DTTPB concentration of 5 μM. The mixtures were incubated at 37 °C for 15 min, and then added into Corning® Costar 96-Well Black-Bottom Plates (100 μL $well^{-1}$). The PL spectra of the mixtures were measured by Molecular Devices SpectraMax i3x Multi-Mode Microplate Detection System. Excitation wavelength: 490 nm, bandwidth: 9 nm.

### 1.4 Bacteria imaging

The staining and imaging of bacteria was described previously [3]. Generally, *S. mutans* were grown in BHI, *S. aureus* and *E. coli* were grown in LB liquid culture medium at 37 °C overnight. The concentration of bacteria was determined by measuring the optical density at 600 nm ($OD_{600}$). The bacteria concentration was then adjusted to an $OD_{600}$ value of 0.6 by diluting the bacterial suspension with culture medium. Then, 5 mL bacteria were collected and harvested by centrifuging at 10,000 ×g for 1 min. The culture medium was removed and the bacteria were washed twice with PBS and then resuspended in 2 mL DTTPB-containing PBS (dye concentration: 5 μM). The bacteria were incubated at 37 °C for 0.5 h. To remove the unbounded

DTTPB molecules, the stained bacteria were washed 3 times with PBS. The bacteria were imaged under CLSM (Nikon, Eclipse Ti-S, Japan) fitted with a 100× oil immersion objective lens and processed using NIS-Elements AR software. Excitation wavelength: 561 nm, red channel: 620–720 nm.

**1.5 Killing test of planktonic bacteria**

*S. mutans* were cultured in BHI medium overnight at 37 °C, harvested and diluted with PBS to an $OD_{600}$ value of 0.6 prior to usage. The culture medium was removed and the bacteria were washed twice with PBS and then resuspended in DTTPB-containing PBS at designated concentrations. The bacteria were incubated at 37 °C for 15 min. Afterwards, DTTPB-containing PBS was removed, and the bacteria were washed twice with PBS and then resuspended in PBS. 50 μL of each sample was added to a 96-well microtiter plate and exposed to white-light irradiation (36 $mW/cm^2$) for 10 min. The bacteria were then subjected to fluorescence staining, plate count method and spot plate assay to evaluate their viability. An OPPLE LED lamp was employed as the source of white light. The luminescence power was adjusted by tuning the height of the white light source and measured by a Compact Power and Energy Meter Console (PM100D, Thorlabs) together with a Microscope Slide Power Meter Sensor Head (S170C, Thorlabs)

For fluorescence staining, the culture was stained with Live & Dead™ Viability/Cytotoxicity Assay kit following manufacture instructions. Bacteria cells were imaged with an CLSM (Nikon, Eclipse Ti-S, Japan). 488 nm laser and 515 – 550 nm emission filter were used for the green channel, while 561 nm laser and 570 – 620 nm emission filter were used for the red channel.

For plate count method, 100 μL of bacterial suspension diluted by an appropriate multiple with PBS was plated on BHI agar and subjected to plate count method for evaluation of bacteria viability. Bacterial viability was expressed as the ratio of the colony forming unit (CFU) from sample treated with DTTPB to the CFU from sample treated with PBS. Representative plates were imaged with a Bio-Rad Universal Hood

II- GelDoc System.

For spot plate assay, the bacteria solution was serially diluted by designated multiples. Afterwards, we aseptically pipetted 3 separated spots onto the BHI agar plate using 10 μL of each diluted solution. The bacteria were then cultured at 37 °C overnight. The spots were imaged with a a Bio-Rad Universal Hood II- GelDoc System.

In order to study the subtle morphological changes, scanning electron microscope (SEM) was employed. First, *S. mutans* was incubated at 37 °C until $OD_{600}$ reached 0.6. The culture medium was removed and the bacteria were washed twice with PBS, and then resuspended in DTTPB-containing PBS at designated concentrations (0 μM, 2 μM, 5 μM, 10 μM). The mixture was incubated at 37 °C for 15 min in the dark and followed by irradiating with white light (36 $mW/cm^2$) for 10 min. Samples were washed with PBS three times immediately and resuspended in 2.5% glutaraldehyde at 4 °C overnight. After washing with PBS three times and dehydrating with ethanol (30%, 50%, 70%, 90% and 100%) successively, and dehydrating with tert-butanol (30%, 50%, 70%, 90% and 100%) successively, samples were dropped on a sterilized cover glass, dried and stored at -80 °C until sputter-coating with gold-palladium 20 mA for 45 s and observed using a Zeiss Sigma 300 scanning electron microscopy.

### 1.6 Biofilm imaging

For biofilm imaging, mid-log phase *S. mutans* cells were harvested and washed three times with PBS. The *S. mutans* cells were resuspended in BHI broth in a confocal dish at an $OD_{600}$ value of 0.6, and then incubated anaerobically for 24 h at 37 °C. The culture medium was removed and biofilms were washed twice with PB. Then, 2 mL of DTTPB-containing PBS (5 μM) was added. After incubation for 0.5 h at 37 °C, biofilms were rinsed three times with PBS. The biofilm was imaged under CLSM (Nikon, Eclipse Ti-S, Japan) fitted with a 60× oil (biofilms) immersion objective lens and processed using NIS-Elements AR software. Excitation wavelength: 561 nm, red channel: 620–720 nm.

## 1.7 Inhibition of biofilm formation

We conducted survival rate assay to study the effect of DTTPB-mediated PDT. *S. mutans* were incubated at 37 °C until $OD_{600}$ reached 0.6. The bacteria were harvested by centrifuging and resuspended in PBS solution. Then 10 μL of the bacteria solution in PBS was allocated into 96-well plate and diluted with 40 μL of DTTPB-containing PBS to reach designated DTTPB concentrations (0 μM, 2 μM, 5 μM, 10 μM). The mixture was incubated at 37 °C for 15 min in the dark, followed by irradiating with white light (36 mW/cm$^2$) for 10 min. Samples were washed with PBS three times immediately. Then, these samples were inoculated in BHI containing 1% (wt/vol) sucrose at 37 °C overnight anaerobically to allow the formation of biofilm.

To quantify biofilm formation, the biofilm was stained by NucGreen for 15 min and imaged with a CLSM (Nikon, Eclipse Ti-S, Japan). 488 nm laser and 515 –550 nm emission filter was used for *S. mutans* biofilm imaging. By analyzing the fluorescence signals from NucGreen with Comstat2, biofilm biomass and biofilm average thickness were evaluated.

Biomass was determined as the volume of all voxels containing biomass divided by the area. Comstat2 counted all voxels above a threshold. The number of voxels was multiplied by the height (z) and width (x) of the voxels and the length (y) in μm (= biovolume). The biovolume is divided by the area of the substratum to obtain a value independent of the size of the observation area. The unit is μm$^3$/μm$^2$. The average thickness was a parameter obtained in the thickness distribution. The thickness distribution demonstrated the height distribution of the biofilm. The height was calculated as the length of the continuous pixels containing the biomass in the vertical direction. The average thickness was the average height of all continuous pixels in the vertical direction containing biomass for the entire viewing area. The unit of the average height was μm.

## 1.8 Oral biofilm model.

In order to establish an *in-vitro* oral biofilm model, biofilms were formed on sterilized

hydroxyapatite (sHA) disks (surface area, 2.7 ± 0.2 $cm^2$ , Kunshan Overseas Chinese Technology New Materials Co., Ltd), vertically suspended in 24-well plates using a custom-made wire disc holder, mimicking the smooth surfaces of the pellicle-coated tooth, as described previously the mouth [4,5]. HA disk was coated with filter-sterilized saliva for 2 h at 37 °C (the saliva was prepared as described previously) [6]. *S. mutans* was grown in BHI at 37 °C anaerobically to mid-exponential phase. Each of these sHA disks were inoculated with 10 µL of *S. mutans* ($OD_{600}$ 0.6) in 1 mL BHI containing 1% (wt/vol) sucrose at 37 °C overnight anaerobically.

*S. mutans* biofilm growing on the sHA disks was harvested and washed, and co-cultured with 20 µM DTTPB at 37 °C for 15 min in the dark. Then the samples were irradiated with white light (36 mW/$cm^2$) for 10 min, and rinsed three times with PBS. Afterwards, samples were treated with 0.12% of chlorhexidine (CHX) for 30 min. Finally, the biofilm was stained by a Live & Dead™ Viability/Cytotoxicity Assay Kit and imaged with a CLSM (Nikon, Eclipse Ti-S, Japan) or preparation for SEM imaging as describe above. At the same time, the untreated biofilms, the biofilms co-cultured with PBS and irradiated were set as control groups, respectively. Three random fields of each sample were chosen for the analysis of bacterial survival rate, biofilm biomass and biofilm average thickness by Comstat2. For calculation of biofilm biomass and biofilm average thickness, fluorescence from both green channel and red channel was employed for the analysis.

**1.9 Measurement of ROS**

10 µL of *S. mutans* ($OD_{600}$ at 0.6) was transferred into a 15-mm confocal dish containing 1 mL BHI broth supplemented with 1% sucrose. Then the dish was incubated at 37 °C anaerobically in the dark for 24 h. The ROS generated in the *S. mutans* biofilm was investigated by staining the biofilm with DCFH-DA (10 µM). In brief, the biofilm was washed with PBS and incubated with 100 µL of DCFH-DA (10 µM) for 15 min in the dark at 37 °C, and washed with PBS again. Then, 100 µL of

DTTPB (20 μM) or PBS was added into the biofilm for incubation for 30 min at 37 °C and irradiated by white light (36 mW/cm$^2$) for 10 min. The biofilm treated with DTTPB but no irradiation was set as the control. At last, biofilm was washed with PBS, and fluorescence was imaged by a CLSM (Nikon, Eclipse Ti-S, Japan).

**1.10 Lactic acid measurement**

For lactic acid measurement, mid-log phase *S. mutans* cells were harvested and washed three times with PBS. The *S. mutans* cells were resuspended in PBS in a 12-well plate at an $OD_{600}$ value of 0.6, and DTTPB was added at final concentrations of 10 μM, incubated for 30 min at 37 °C and then irradiated with white light for 10 min at 36 mW/cm$^2$. Untreated bacteria suspension was used as the negative control and 0.1% DMSO was used as the solvent control. Following anaerobic incubation at 37 °C for 3 h and 8 h, the *S. mutans* cells were removed by centrifugation (10,000 × g, 5 min, 4 °C), and a lactic acid assay kit (E-BC-K044-M, Elabscience) was used to measure the lactate concentrations of the *S. mutans* supernatants. The absorbance at 530 nm ($A_{530}$) was measured using a microplate spectrophotometer, and standard curves were used to calculate the lactate concentrations [7]. Three replicates were performed for each sample.

**1.11 RNA isolation and quantitative real-time PCR (qRT-PCR)**

To study the effect of DTTPB without/with light on targeted genes (*gtfB, gtfC, gtfD, srtA, nox* and *sodA*) expression, total RNA of *S. mutans* treated with different concentrations of DTTPB (0, 2, 5 and 10 μM) was extracted by TRIzol reagent (Thermo Fisher Scientific) according to the manufacturer protocol. The integrity and purity of RNAs were tested using 1.5% agarose gels, NANO DROP2000 (Thermo). cDNA synthesis was performed according to standard protocols. Briefly, 1 μg RNA was reverse-transcribed using an Oligo(dT)15 Primer (Yeasen) and Enzyme Mix(Yeasen). First, 1 μL Oligo(dT)15 Primer (50 μM) and 13 μL $H_2O$ were added into the RNAs with a denaturation step at 65 °C for 5 min, followed by mixing evenly

with the reverse transcription reaction mixture as follows: 4 μL 5× reaction buffer and 2 μL Enzyme Mix. The reverse transcription reaction was then performed at 25°C for 5 min, 42 °C for 30 min and enzyme deactivation at 85 °C for 5 min.

Primers of qRT-PCR were designed with Primer Premier 6.0 software (Primer, Canada). The expression level of target genes related to the biofilm formation was measured by qRT-PCR. The primer sequences are listed in **Table S1**. Each qRT-PCR reaction mixture contained: 5 μL first-strand cDNA from the RT-PCR, 10 μL Taq Pro Universal SYBR qPCR Master Mix (Vazyme), 0.5 μL of each primer (25 μM), and 4 μL $H_2O$ to a final volume of 20 μL. The amplification reaction was achieved through one denaturation cycle at 95 °C for 3 min followed by 40 cycles at 94 °C for 5 s, 60 °C for 30 s and a final extension cycle at 72 °C for 20 s in the CFX96TM RT-PCR detection system (BIO-RAD).

Three independent repetitions of each sample were performed for the test. 16S rRNA was amplified as an internal standard to normalize gene expression and results were analyzed with the $2^{-\Delta\Delta Ct}$ method. All assays were performed with three independent biological replicates. Data analyses were performed using Rotor-Gene 4.6.

**1.12 Biofilm eradication test**

In order to study the effect of DTTPB and white light irradiation on *S. mutans* biofilm eradication, bacterial Live/dead staining, SEM imaging as well as crystal violet (CV) assay were applied [8]. Briefly, a *S. mutans* biofilm grown for 24 hours on the 15-mm confocal dishes was washed with PBS, incubated with 20 μM of DTTPB for 15 min at 37 ℃and then irradiated with white light (36 mW/cm$^2$) for 10 min. Afterwards, DTTPB was removed and the bacteria were washed three times with PBS. After incubation with CHX for 30 min, samples were washed with PBS three times and treated with a live & dead™ viability/cytotoxicity assay kit (US Everbright® INC.). These biofilms were then imaged by a CLSM (Nikon, Eclipse Ti-S, Japan) or prepared for SEM imaging as described above. 488 nm laser and 515 – 550 nm emission filter were employed for green channel, while 561 nm laser and 570 – 620

nm emission filter were used for the red channel.

For CV assay, *S. mutans* biofilm grown in 96 well microtiter plate. After anaerobic incubation for 24 h, the planktonic bacteria were gently decanted with PBS three times. Biofilms were then fixed with methanol for 15 min and colored with 0.1% crystal violet staining (CV, Hushi, shanghai, China) for 5 min [2]. The excess dye was removed with PBS for three times, and biofilms were decolored with 30% acetic acid for 30 min at 37°C to dissolve evenly. The absorbance was determined at 595 nm with a microplate spectrophotometer.

### 1.13 Discoloration test of DTTPB on sHA

HA dishes were prepared for *in-vitro* teeth model whitening assay. HA were pretreated with artificial saliva for 24 hours to form an acquired film (2). The specimens were randomly divided into three groups (n = 3), and exposed to different staining beverages (cola, coffee, and black tea) for 7 consecutive days. The exposure treatments were refreshed every 24 h with new staining drinks. After the process of coloration, running water was used to remove the residual substance that was not firmly adhered to the enamel surface. Every group was treated without/with 20 μM of DTTPB, and storage in dark or irradiation with white light (36 mW/cm$^2$) and photographed at 0 min, 10 min, 30 min and 60 min with a DSLR camera (Canon EOS 700D with EF-S 18-135mm IS STM Lens).

### 1.14 Teeth discoloration test of DTTPB

Intact and noncarious teeth (including smoke-stained teeth) in diverse locations were collected from Yanling Taocheng health center, Henan, and informed written consent was obtained from all participants or next of kin. The teeth were immersed in saturated 0.1% thymol solution, and stored at 4 °C until usage. This study was approved by the Medical Ethics Committee, School of Medicine, Wuhan University (No. WHU2021-YXY00566).

Every group was treated without/with 20 μM of DTTPB, and storage in dark or

irradiation with white light (36 mW/$cm^2$) and photographed at 0 min, 10 min, 30 min and 60 min with a mirrorless camera (Canon EOS M5 with EF-M 18-150 mm f/3.5-6.3 IS STM Lens).

**1.15 Hemolysis test**

Fresh mouse blood were added heparin sodium, collected by centrifugation, and then resuspended in PBS (20%, *v*/*v*). 100 μL mouse red blood cell suspension were mixed with 300 μL of DTTPB to reach designated concentrations, and co-cultured for 2 h at 37°C. The mixture was centrifuged (2000 ×g, 10 min) to remove red blood cells. The absorbance was measured at 540 nm. Deionized water and 5% $H_2O_2$ were used as control groups. The hemolysis ratio (%) was calculated as follows:($OD_{540}$ sample/$OD_{540}$ deionized water) × 100%.

**1.16 Cytotoxicity assay**

For Calcein AM/PI staining assay, cells were co-clutured with 5% $H_2O_2$ or different concentrations of DTTPB (0, 5, and 20 μM) for 0.5 and 2 h. After rinsing with PBS, the cells were incubated with the Calcein AM/PI staining according to the protocol and imaged with a CLSM (Nikon, Eclipse Ti-S, Japan). 488 nm laser and 500 – 550 nm emission filter were employed for the green channel. 561 nm laser and 570 – 620 nm emission filter were used for the red channel.

**1.17 Statistical methods**

The images and fluorescence intensity were processed or analyzed by Image J and COMSTAT2.0. All of the statistical analyses were performed using GraphPad Prism8.0. Experimental results were presented with mean± standard deviation (SD). The statistical significance of differences was determined by a paired two-tailed t-test using Origin software. $p < 0.05$ (*), $p < 0.01$ (**), and $p < 0.001$ (***) were used to represent statistical differences.

For Lab system, the lightness value, *L*, defines black at 0 and white at 100. The *a* and *b* are in the range of −128 to 127. The lightness percentage, a and b value are

quantized to 255. In order to get the lab chrominance values, lightness byte value is divided by 2.55 and the *a* and *b* byte values are subtracted 128[9].

## 2. Supplementary Figures

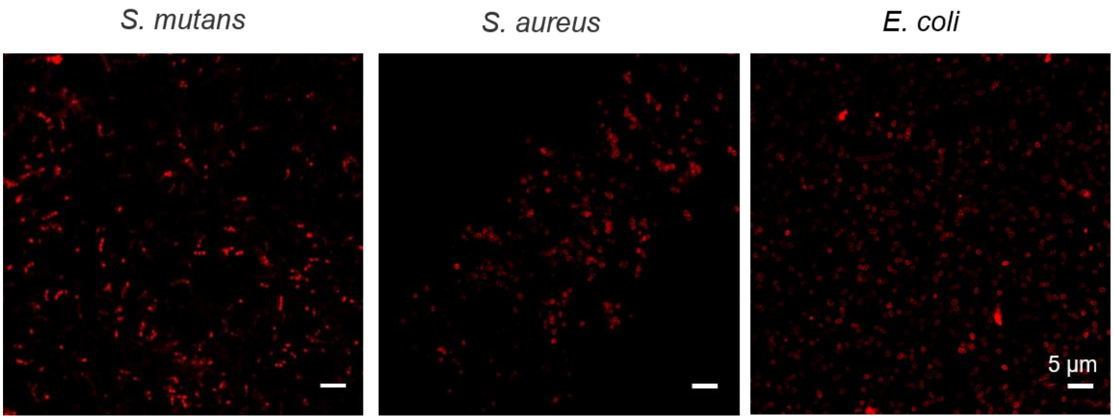


**Figure S1**. CLSM images of *S. mutans, S. aureus* and *E. coli* incubated with 5 µM of DTTPB. 561 nm laser and 620 – 720 nm emission filter were used for imaging.

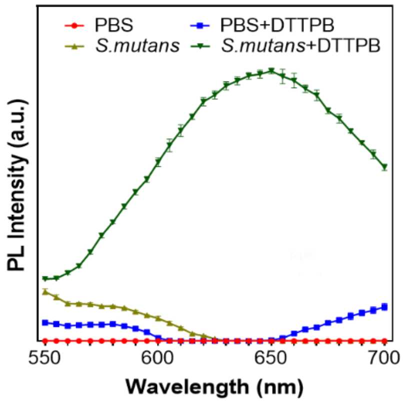


**Figure S2**. PL spectra of *S. mutans* incubated without/with 5 μM of DTTPB in PBS solution. Excitation wavelength: 490 nm, bandwith:9 nm.

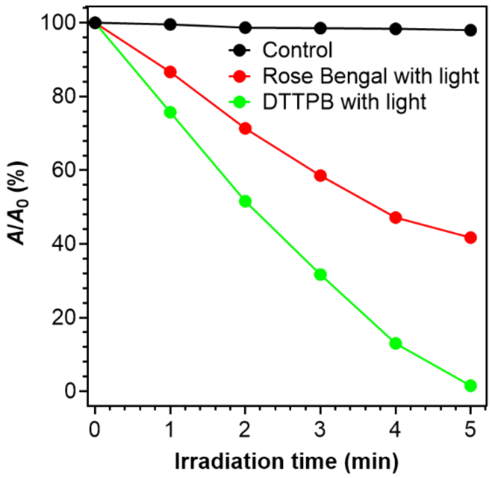


**Figure S3**. Evaluation of DTTPB-sensitized ROS production with ABDA. Rose Bengal was employed for comparison. Decomposition rates of ABDA in the absence or presence of DTTPB under light irradiation (36 mW/cm$^2$), where $A_0$ and $A$ are the initial and final absorbance of ABDA at 378 nm, respectively.

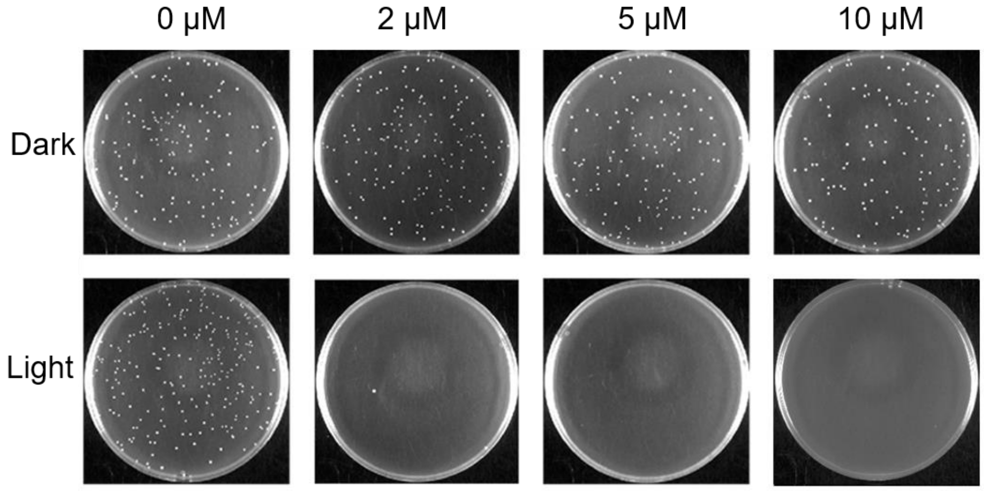


**Figure S4**. Images of BHI agar plates employed for quantification of *S. mutans* viability. The *S. mutans* were treated with varied concentrations of DTTPB prior to quantification.

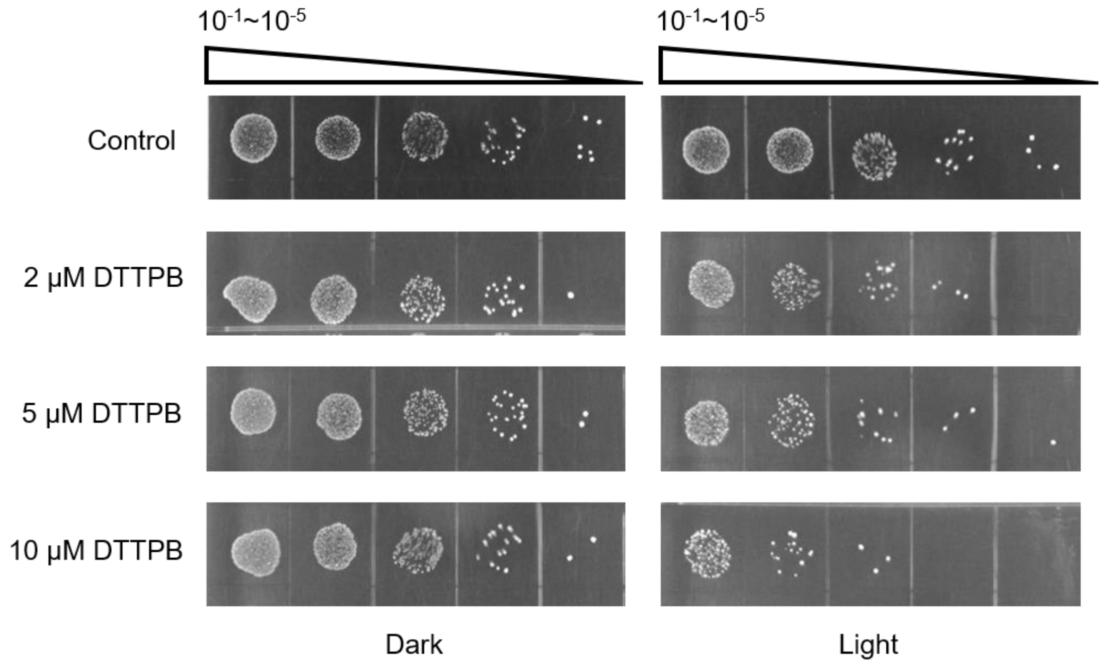


**Figure S5**. The viability of *S. mutans* evaluated by spot plate assay. *S. mutans* were incubated with varying concentrations of DTTPB under white-light (36 mW/cm$^2$) irradiation.

**Table S1**. Primers used in quantitative real-time PCR for detecting *gtfB*, *gtfC*, *gtfD*, *srtA*, *nox*, *sodA* levels.

| **qRT-PCR primers** | **Fp (5'to 3')** | **Rp (5'to 3')** |
|---|---|---|
| *gtfB* | TGCTCCAAATTGCTGGGGAT | CGTTGTCACTCCATGCCTCT |
| *gtfC* | CGTCTGTCCGCTATGGTAAAG | GGTCAAGAGTAAAGGTCGGTAAG |
| *gtfD* | TGCAAGCGACGGAAAACAAG | GCCTGTCAGAGCTTCACCAT |
| *srtA* | TGGCAATTCCGCCAATTACAG | AAGGCTGCCCATTCTTCCTT |
| *nox* | CGCTCTTCGCTCAGGTATTGT | CGCTTTGCTTTTTCTTGCGTT |
| *sodA* | GCTCAGGTTGGGCTTGGTTA | AGCGTGTTCCCAGACATCAA |
| *16S rRNA* | CCTACGGGAGGCAGCAGTAG | CAACAGAGCTTTACGATCCGAAA |

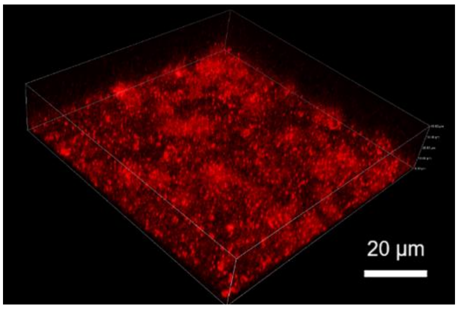


**Figure S6**. CLSM images of *S. mutans* biofilm incubated with 5 µM of DTTPB. 561 nm laser and 620 – 720 nm emission filter were used for imaging.

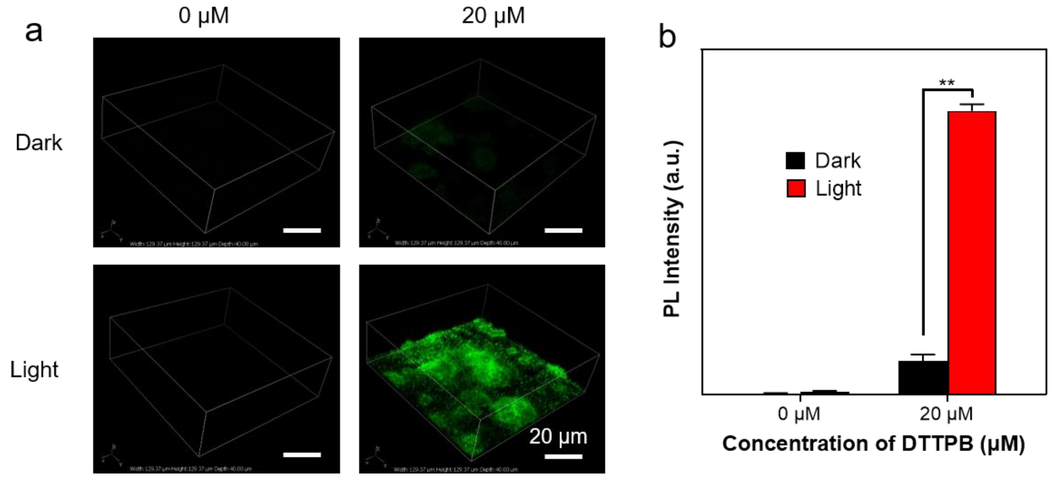


**Figure S7**. Evaluation of ROS production in biofilm. (a) Confocal 3D images of biofilms and (b) quantification of the corresponding fluorescence intensity according to 5 random sights of *S. mutans* biofilms by COMSTAT. *S. mutans* biofilms were incubated with DCFH, without/with 20 μM of DTTPB and white-light (36 mW/cm$^2$) irradiation. Data are shown as mean ± standard deviation.

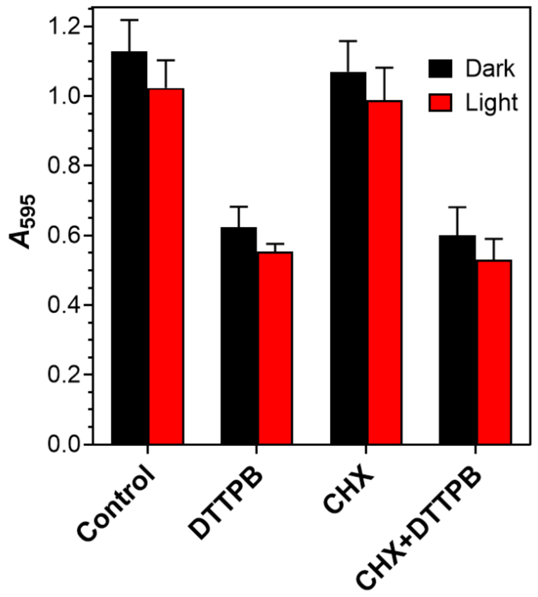


**Figure S8.** Evaluation of the effect of DTTPB and CHX on exsiting biofilm by Crystal Violet staining. Biofilms were fixed with methanol for 15 min and stained with 0.1% crystal violet staining for 5 min. The excess dye was removed with PBS for three times, and biofilms were decolored with 30% acetic acid for 30 min at 37°C to dissolve evenly. The absorbance was determined at 595 nm with a microplate spectrophotometer. Data are shown as mean ± standard deviation with three replications.

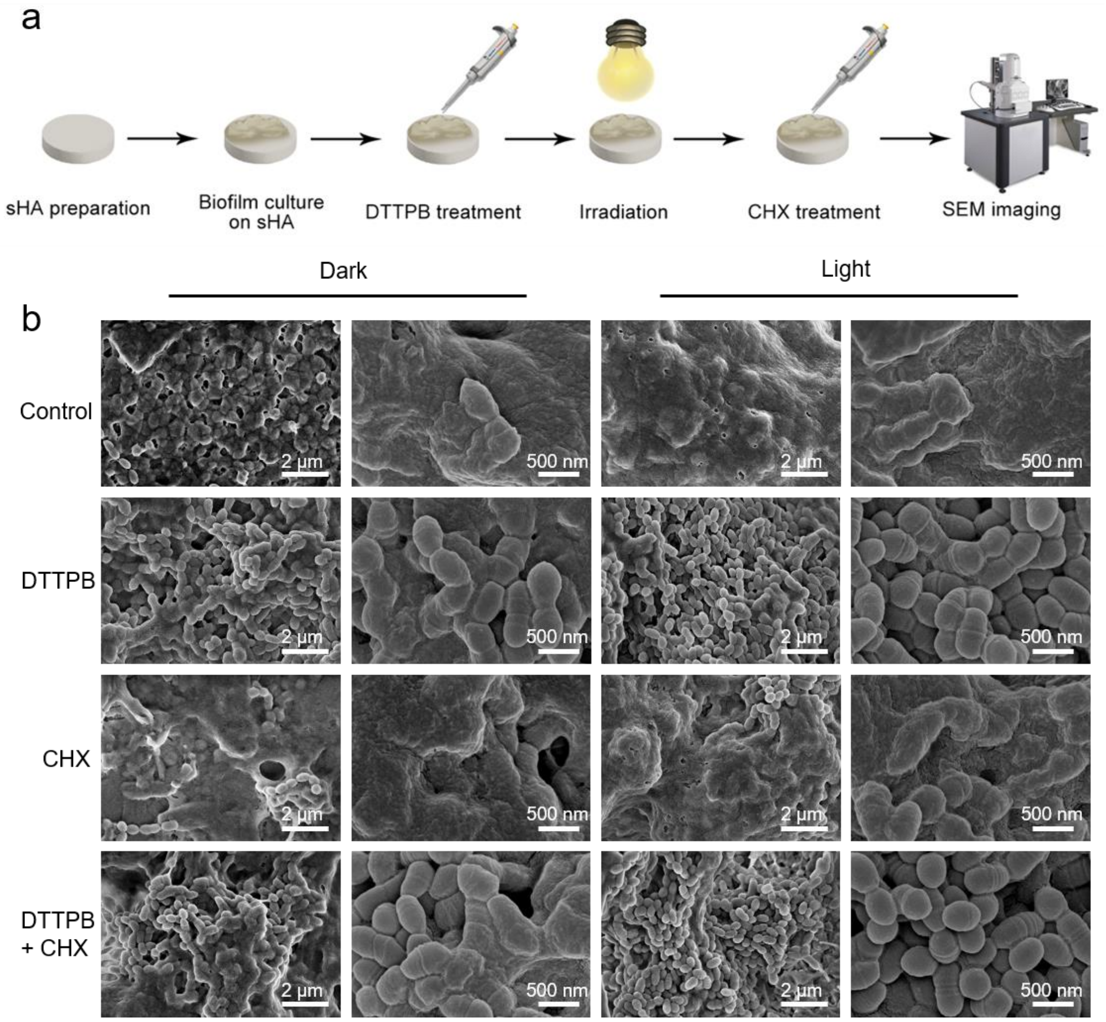


**Figure S9**. (a) Schematic illustration of experimental processes for determining the morphology changes of *S. mutans* biofilms after combined treatment with DTTPB and CHX on sHA. (b) SEM images of *S. mutans* biofilms. 1-day-old *S. mutans* biofilms on sHA were cultured without/with 20 μM of DTTPB and white-light irradiation for 10 min (36 mW/cm$^2$), and treated without/with 0.12% CHX for 30 min.

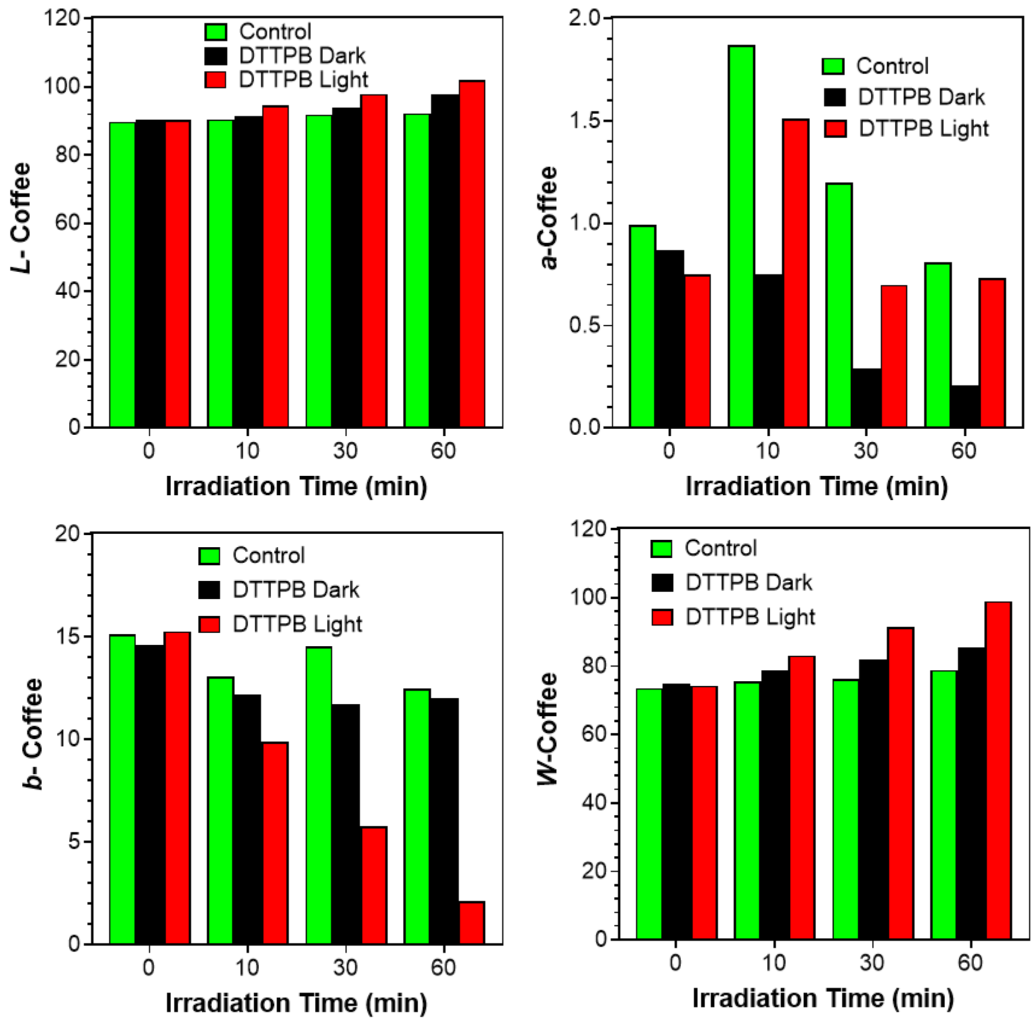


**Figure S10**. *L*, *a*, *b*, *W* of coffee-colored sHA treated with 20 μM of DTTPB in the absence/presence of white-light irradiation (36 mW/cm$^2$).

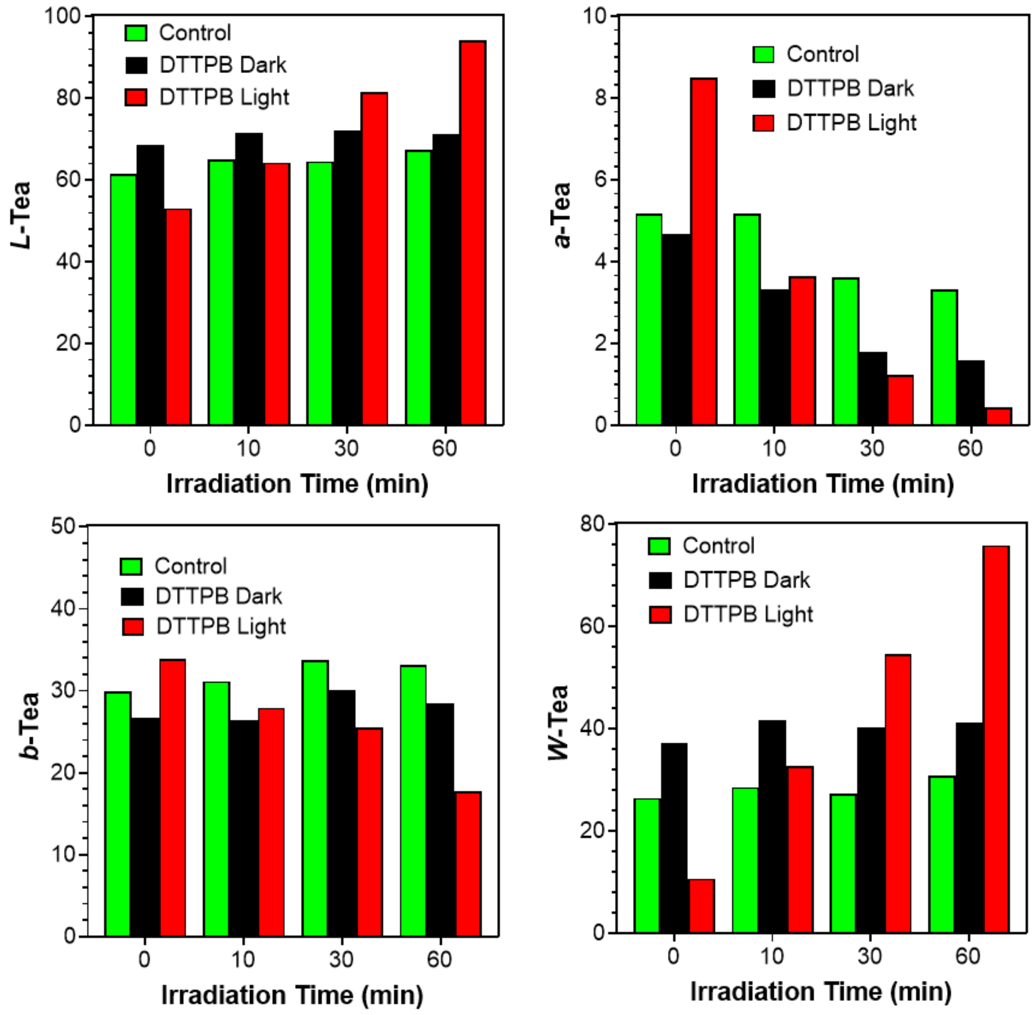


**Figure S11**. *L*, *a*, *b*, *W* of tea-colored sHA treated with 20 μM of DTTPB in the absence/presence of white-light irradiation (36 mW/cm$^2$).

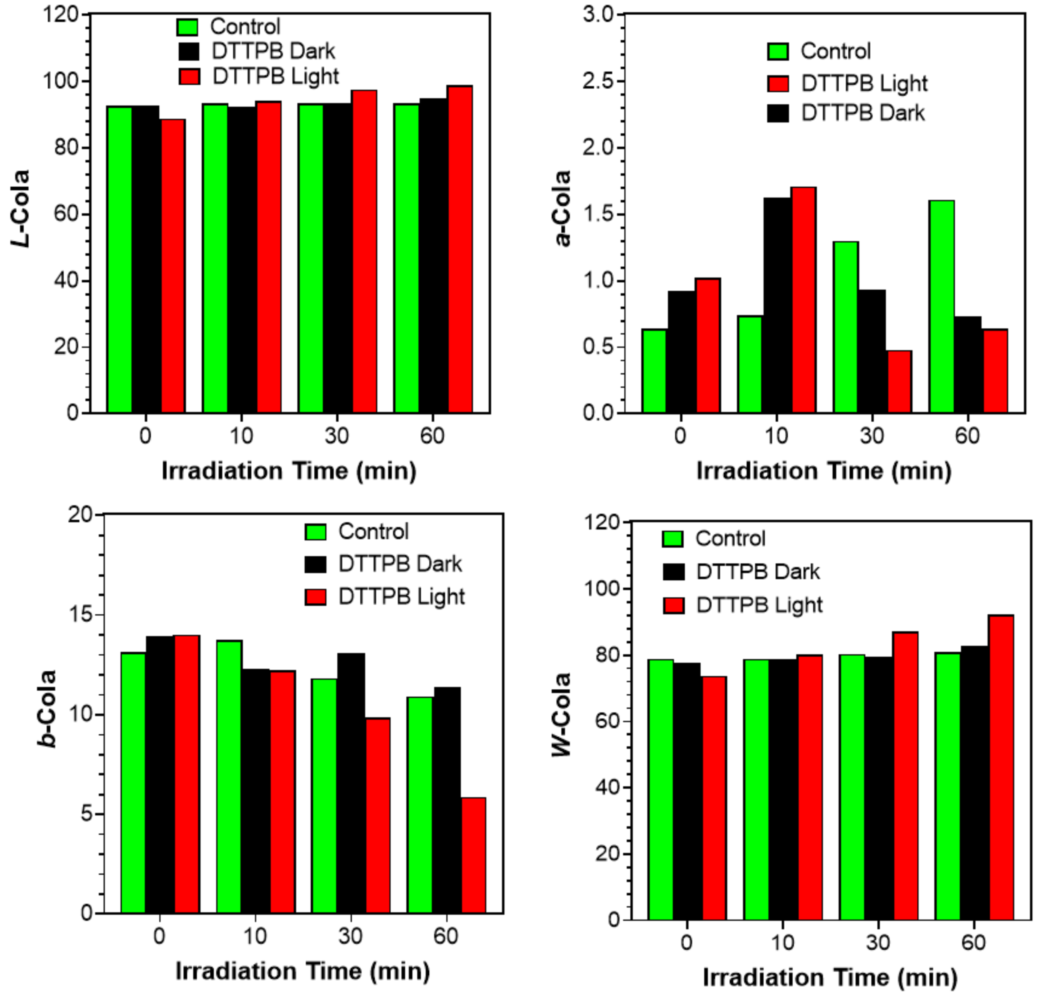


**Figure S12**. *L*, *a*, *b*, *W* of cola-colored sHA treated with 20 μM of DTTPB in the absence/presence of white-light irradiation (36 mW/cm$^2$).

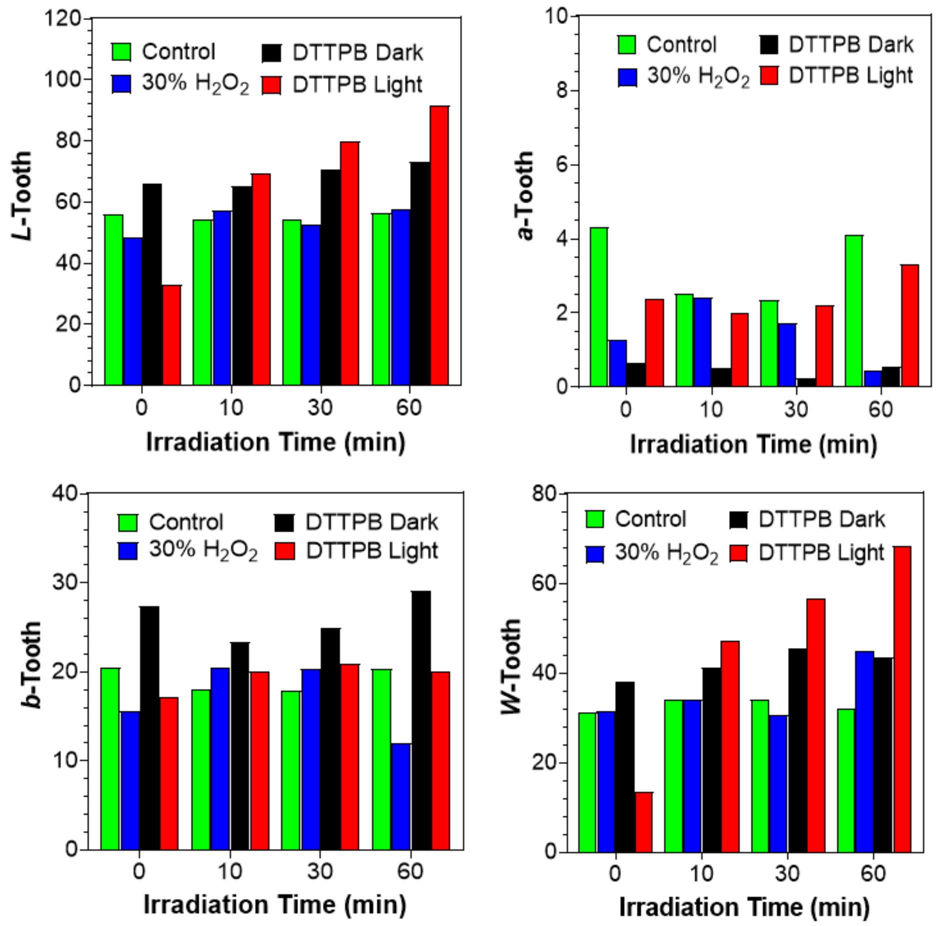


**Figure S13**. *L*, *a*, *b*, *W* of colored intact and noncarious teeth treated with 20 μM of DTTPB and white-light irradiation (36 mW/cm$^2$) or 30% $H_2O_2$.

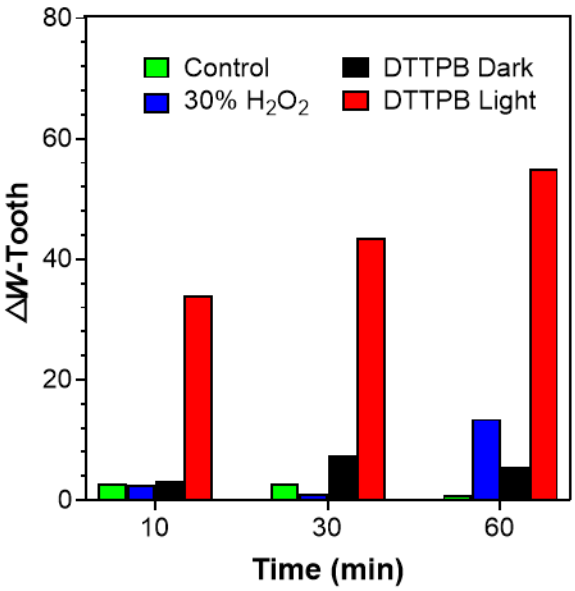


**Figure S14**. Δ*W* of colored intact and noncarious teeth treated with 20 μM of DTTPB and white-light irradiation (36 mW/cm$^2$) or 30% $H_2O_2$.

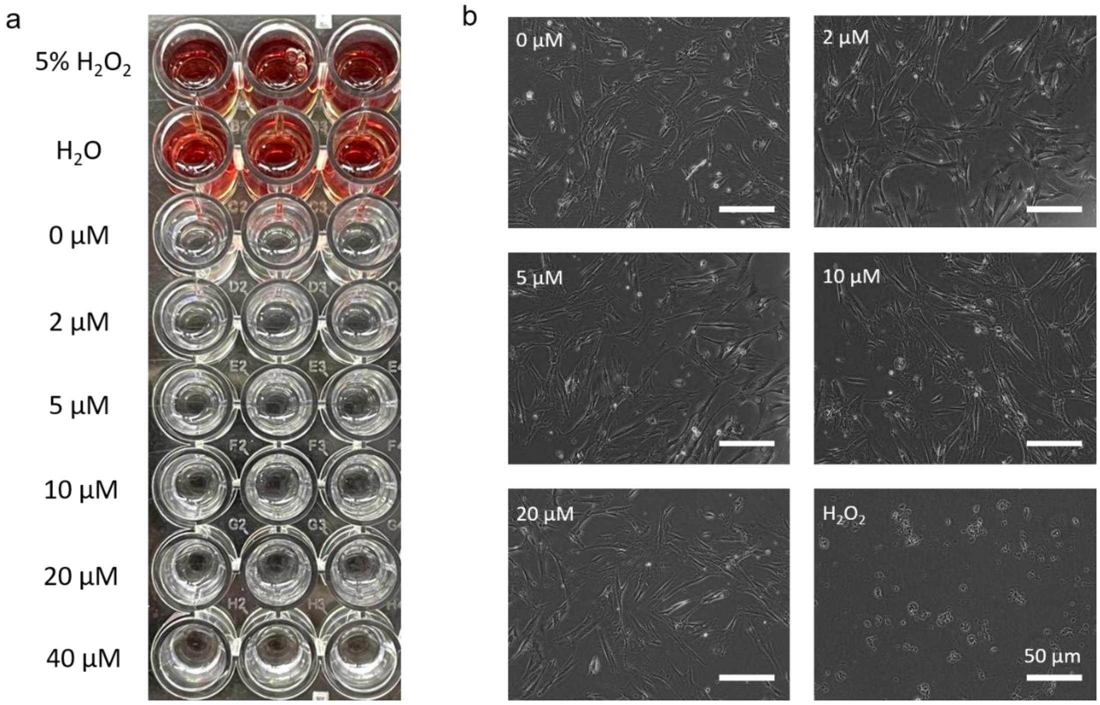


**Figure S15**. (a) The picture indicates that no hemolysis caused by different concentrations of DTTPB for 2 h. (b) Morphology of MRC-5 cells co-cultured with different concentrations of DTTPB (0, 2, 5, 10, 20 μM) and 5% $H_2O_2$ for 2 h.

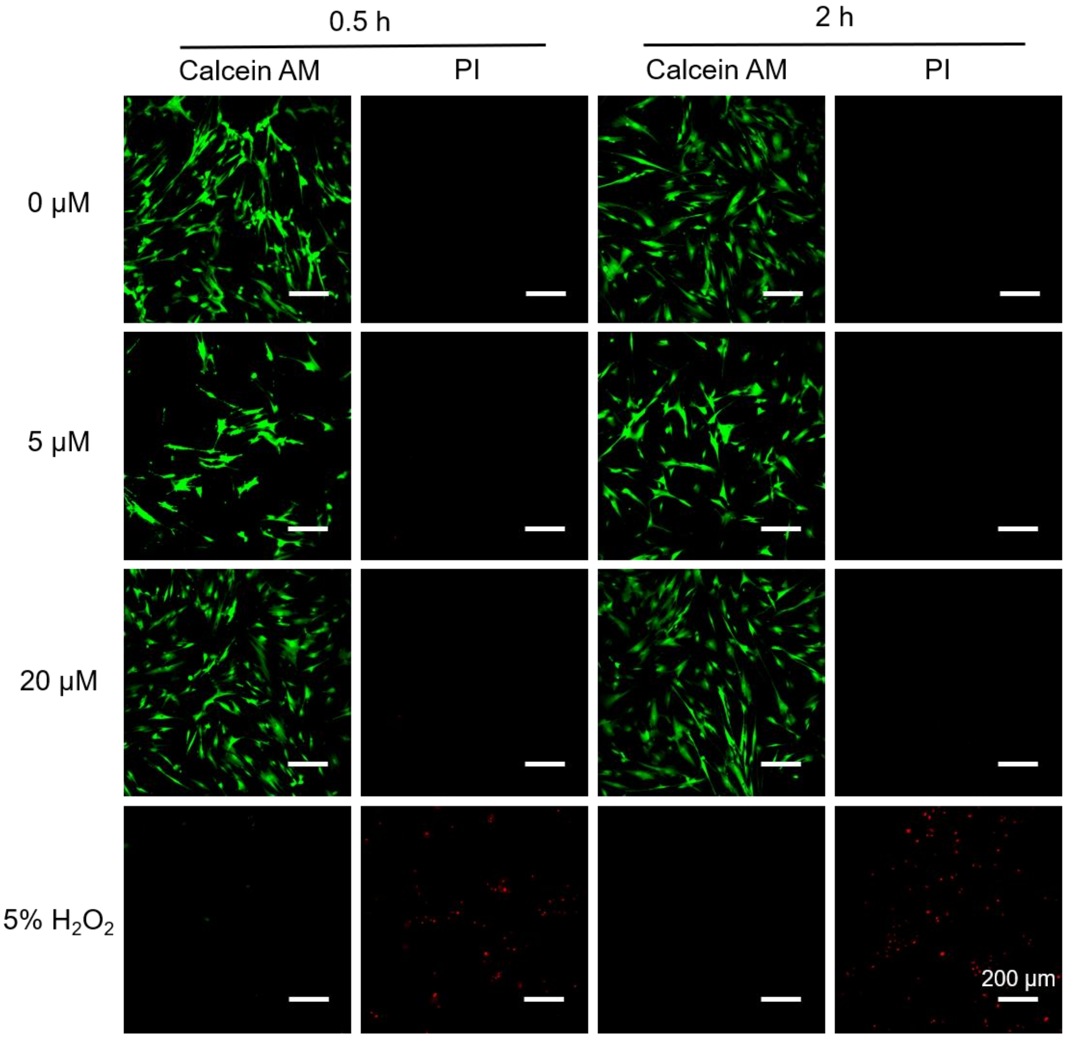


**Figure S16**. Evaluation of the toxicity of DTTPB on cells. MRC-5 cells were incubated with different concentrations of DTTPB or 5% $H_2O_2$, stained with Calcein AM/PI, followed by imaging with a CLSM. 488 nm laser and 500 – 550 nm emission filter were employed for the green channel. 561 nm laser and 570 – 620 nm emission filter were used for the red channel.

## Reference

[1] R. Tao, Z. Tong, Y. Lin, Y. Xue, W. Wang, R. Kuang, P. Wang, Y. Tian, L. Ni, *Peptides* **2011**, *32*, 1748.

[2] Y. Niu, K. Wang, S. Zheng, Y. Wang, Q. Ren, H. Li, L. Ding, W. Li, L. Zhang, *Antimicrob Agents Chemother* **2020**, *64*, e00251.

[3] M. Gu, Z. Zeng, M. Y. Wu, J. K. Leung, E. Zhao, S. Wang, S. Chen, *Chem. Asian J.* **2019**, *14*, 775.

[4] W. F. Liljemark, C. G. Bloomquist, G. R. Germaine, *Infect. Immun.* **1981**, *31*, 935.

[5] J. Xiao, M. I. Klein, M. L. Falsetta, B. Lu, C. M. Delahunty, J. R. Yates, A. Heydorn, H. Koo, *PLoS Pathog.* **2012**, *8*, 7.
[6] L. D. Ridgway, M. D. Wetzel, D. Marchetti, *Exp. Ther. Med.* **2011**, *2*, 229.
[7] Y. Wang, X. Wang, W. Jiang, K. Wang, J. Luo, W. Li, X. Zhou, L. Zhang, *J. Oral Microbiol.* **2018**, *10*, DOI 10.1080/20002297.2018.1442089.
[8] D. Hu, Y. Deng, F. Jia, Q. Jin, J. Ji, *ACS Nano* **2020**, *14*, 347.
[9] https://magnetiq.ca/pages/acb-spec/
